# Application of an Interactive, Hands-On Nutritional Curriculum for Pediatric Residents

**DOI:** 10.1097/PG9.0000000000000384

**Published:** 2023-11-13

**Authors:** Cory Wyatt Jones, Andrew A.M. Singer

**Affiliations:** From the * Department of Pediatrics, University of Michigan; † Department of Pediatrics, University of South Carolina, SC.

**Keywords:** education, community, didactic

## Abstract

Currently, there exists a scarcity of suitable nutrition training resources for the primary care physician (PCP) and a paucity of educational materials for pediatric residency programs. Barriers to nutritional education include: a lack of well-defined competencies, a dearth of centralized resources for nutritional education, and a reliance on didactic teaching methodology. Because PCPs often cite a lack of confidence as a primary reason for not providing nutritional counseling, we created an interactive 3-pronged nutritional curriculum for pediatric residents with the aim of increasing their confidence to provide nutritional counseling to patients. This curriculum included an in-person visit to a local supermarket, an online, interactive case during the resident’s continuity clinic, and an interactive lecture. There was a statistically significant change in pediatric residents’ confidence to manage issues of outpatient nutrition management. We find this particularly relevant as increasing physician confidence is key to increasing nutritional counseling in a clinical setting.

**What Is Known?**

- Primary care physicians cite uncertainty as a reason for not pursuing nutritional counseling in the clinical setting.
- Primary care physicians consistently outperform their self-reported confidence on objective evaluations of nutritional knowledge.
- Hands-on nutritional training is superior to didactic training.

**What Is New?**

- Demonstration of the use of a semi-interactive curriculum to increase nutritional confidence among pediatric residents.
- Use of a curriculum with a novel design to improve physician confidence as opposed to a curriculum designed to improve physicians’ fund of knowledge.

## INTRODUCTION

Despite an increase in obesity-related disease in the United States, many primary care providers do not feel confident in the prescription of nutritional therapies for lifestyle modification. Feelings of inadequate preparation to prescribe medical nutritional therapy have been shown in students, residents, fellows, and practicing physicians (1–4). Importantly, Vetter et al (5) demonstrated no correlation between residents’ self-perceived proficiency and their objective knowledge base (5). In a separate study, practicing physicians were given an objective test of their funds of knowledge, on which they scored 70% on average. However, on a Likert scale of 1 (poor) to 5 (excellent) their self-reported knowledge score was only 2.51 on average (6). There are several barriers that lead to these self-perceived deficiencies. In a survey conducted by Huang et al (7) physicians reported “lack of skills in providing brief counseling” and “insufficient knowledge of best clinical practices” as reasons for not pursuing nutritional counseling in the clinical setting. Cumulatively, these studies suggest that patient care may potentially improve by improving the provider’s sense of proficiency without needing to significantly bolster their fund of knowledge.

How to improve physician’s nutritional confidence and competence has been a subject of discussion for many decades (8–10). The lack of well-defined nutritional competencies has been cited by multiple authors (11–13). The American Academy Family Physicians has produced a lengthy document of well-defined nutritional competencies for the practice of family medicine (14). Comparatively, the American Academy of Pediatrics has no official nutritional competencies while the American Board of Pediatrics has produced what they refer to as the “detailed content outline” (15), which in their entirety read as follows: “(1) Newborn and infant feeding, (2) Age-specific nutritional needs, (3) Patient population-specific nutritional needs (eg, vegetarian and vegan), (4) Condition-specific nutritional needs (eg, food allergies and hypertension), and (5) Obesity.” Such vague learning objectives are of limited use to the educator.

The next barrier to nutritional education is the lack of a clear medical home for the management of nutritional-related diseases. Providers often assisting with these diseases include the primary care physician (PCP), pediatric gastroenterologist, and pediatric endocrinologist, among others (16). The variety of providers combined with the lack of centralized resources described above makes it difficult for the creation of robust nutritional education for pediatric residents. Fortunately, the North American Society for Pediatric Gastroenterology, Hepatology, and Nutrition defines one of its central missions “to be a world leader in research, education, clinical practice and advocacy for pediatric gastroenterology, hepatology and nutrition in health and disease” (17). This places the pediatric gastroenterologist as a central cog in the wheel of nutrition education. The pediatric gastroenterologist often has a multitude of resources, such as registered dieticians and GI psychologists, which are not readily available to the PCP.

Another barrier is that at some institutions pediatric nutritional education is limited to a didactic setting despite the fact that nutritional education may be more effectively delivered by interactive, hands-on learning (11). Other authors have focused their interactive nutritional curricula on medical students. The SARS-CoV-2 pandemic accelerated virtual learning and has made it difficult to continue hands-on learning (18).

## METHODS

We sought to implement an interactive curriculum throughout the 3-year course of pediatrics residency. Our residency program is a program of approximately 25 residents per year at the University of Michigan, a tertiary academic facility. All pediatric residents in postgraduate years (PGY) 1–3 were included in our study. An IRB exception was obtained for this study (HUM00175605, University of Michigan).

We began by conducting a needs assessment of nutrition education in our pediatric residency. Residents felt as though they lacked the knowledge, time, and motivation to counsel patients regarding a healthy diet and exercise. This agreed with what had previously been reported (5). A formal needs assessment was undertaken in which we compared the recommendations of the American Academy Family Physicians, American Academy of Pediatrics, and the Nutrition Academic Award Program to the curriculum at our residency. The details of this formal needs assessment can be found in Supplemental Digital Content 1, http://links.lww.com/PG9/A140 entitled “Curriculum Comparisons”. We then developed a curriculum with the goal of providing resources to build pediatric resident confidence in the evaluation, counseling, and management of patients with nutritional needs. We attempted to create a curriculum that allowed residents the opportunity to practice nutritional counseling using an experiential approach. Residents participated in 3 distinct educational modules.

The first module consisted of an in-person visit to a local supermarket. This was done with residents who were currently participating in a required 1-month community health-based rotation. The group was led by one of the pediatric gastroenterology fellows. Groups of 2–4 residents, made up of PGY 1–3, went to the grocery store at a time. While there, residents compared the nutritional content of various foods, discussed barriers to healthy eating, created meal plans, and discussed successful lifestyle modifications. See Supplemental Digital Content 2, http://links.lww.com/PG9/A141 titled “Outline for Grocery Store Tour,” Supplemental Digital Content 3, http://links.lww.com/PG9/A142 titled “Store Handout, and Supplemental Digital Content 4 http://links.lww.com/PG9/A143 titled “Store Handout Answers.” Due to unanticipated restrictions caused by the SARS-CoV-2 pandemic, not all residents were able to complete this activity.

The second module consisted of an online, interactive case during the resident’s continuity clinic. This module focused on finding online resources to help pediatricians manage outpatient nutritional challenges. The students navigated the MyPlate.org website, The American College of Sports Medicine website (acsm.org), the MyFitnessPal mobile app, and the natural medicines website (naturalmedicines.therapeuticresearch.com), all available through the university. For a full outline of the activity, please see Supplemental Digital Content 5, http://links.lww.com/PG9/A144 labeled “OP Module.”

The final module consisted of an interactive lecture. During the curricular development phase and before the pandemic, this was designed to be a face-to-face activity as part of our residency’s traditional noon lecture. However, due to local restrictions, the interactive portions of the lecture were altered to fit an interactive digital format. To make the lecture interactive, the curriculum development team used audience response software and provided the resident team rooms with samples of healthy eating behavior during the lecture. For full details and outlines used in this module please see Supplemental Digital Content 6, http://links.lww.com/PG9/A145 entitled “Outline for Lecture,” Supplemental Digital Content 7, http://links.lww.com/PG9/A146” entitled “Portions Quiz,” and Supplemental Digital Content 8, http://links.lww.com/PG9/A147 entitled “Nutrition Lecture.” The residents were also given the “Rate Your Plate” activity previously published by Craven et al (19).

Concerning data collection, residents were surveyed before preintervention, midintervention, and postintervention. Surveys were taken using Qualtrics. Survey questions were not validated, as no validated measure of physician confidence exists. However, questions were beta-tested on residents, fellows, nurses, and attendees to ensure the clarity of the questions. The entire survey is available under Supplemental Digital Content 9, http://links.lww.com/PG9/A148 entitled “Survey.” Survey questions focused on residents’ confidence to provide outpatient nutritional counseling. Surveys are based on a 5-point Likert scale, gauging level of confidence with 1 representing extremely unconfident, 2 somewhat unconfident, 3 neither confident nor unconfident, 4 somewhat confident, and 5 extremely confident. There were 79 pediatric residents in the preintervention survey and 75 pediatric residents in the midintervention and postintervention survey.

Statistical analysis was performed using Microsoft Excel 2016. As our data consisted of numerical responses that were nonparametric, we used the Wilcoxon matched-pairs signed rank sum test (20,21).

## RESULTS

Twenty-six PGY-1, 24 PGY-2, and 29 PGY-3 were included in the preintervention cohort. We excluded 4 residents due to matriculation, leaving 79 pediatric residents in the preintervention survey and 75 pediatric residents in the midintervention and postintervention survey. Thirty-two (43%) residents completed the in-store activity. Thirty-five residents (47%) completed the lecture portion of the curriculum.

The demographics of the residents who completed the survey are outlined in Table 1. Overall, 61% of residents filled out the preintervention survey with 43% of residents who filled out the midintervention and postintervention surveys.

**TABLE 1.** Survey completion demographics

	Pre-N	Pre-T	Pre%	Mid-N	Mid-T	Mid-%	Post-N	Post-T	Post-%
PGY-1	22	26	85	13	26	50	14	26	54
PGY-2	11	24	46	12	24	50	10	24	42
PGY-3	15	29	52	7	25	28	8	25	32
Total	48	79	61	32	75	43	32	75	43

The demographics of the residents who completed the survey. The absolute number (N) of residents who completed the survey is noted in each column by “Pre-N,” “Mid-N,” and “Post-N.” The total (T) number of residents in each class is noted by “Pre-T,” “Mid-T,” and “Post-T.” The relative percentages are notated by “Pre-%,” “Mid-%,” and “Post-%” for the pre-, mid-, and post-intervention services. 29 PGY-3 were included in the preintervention cohort and 25 PGY-3 were included in the mid- and post-cohort. N = number of residents participating; PGY = postgraduate year.

**FIGURE 1.**
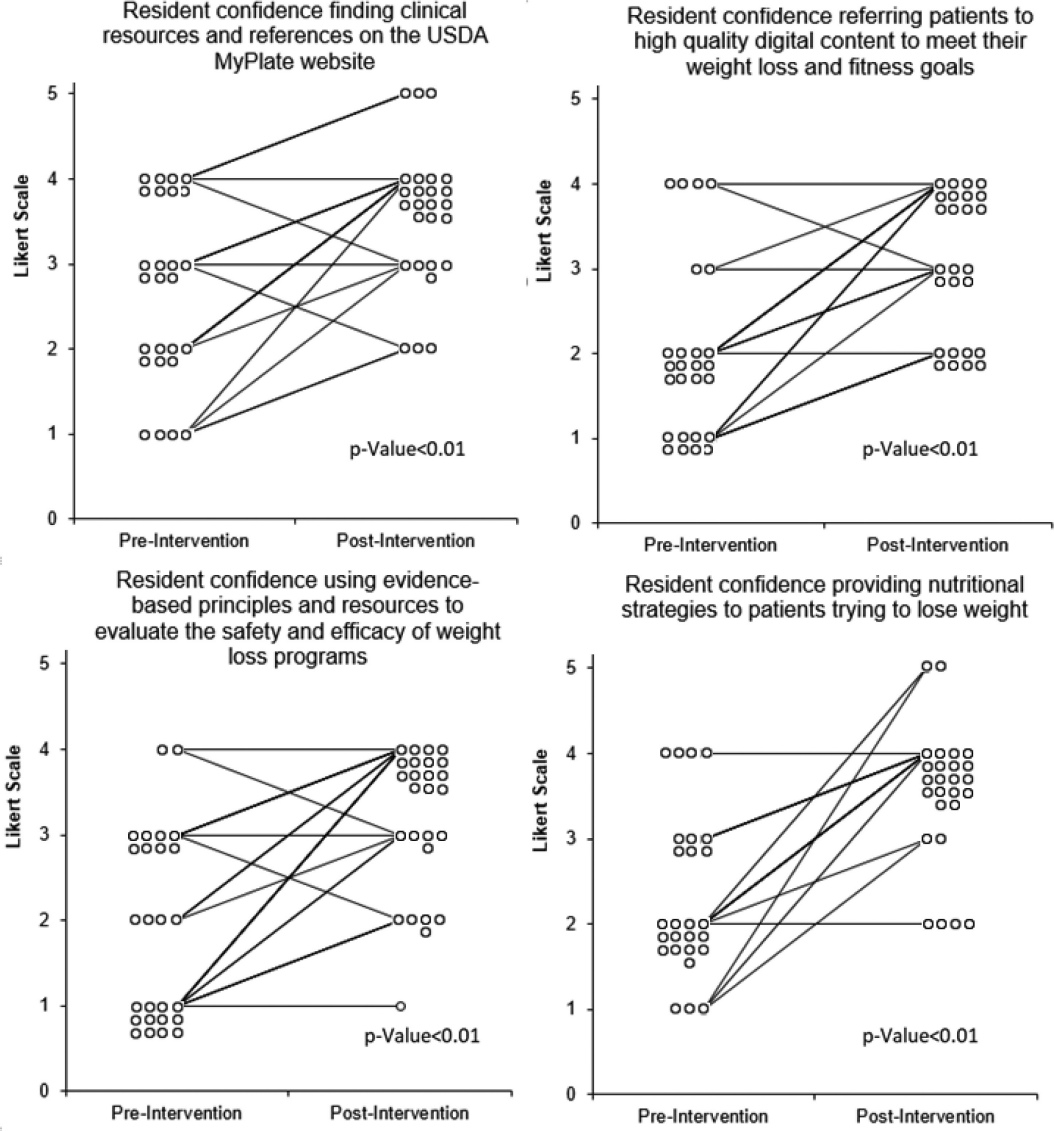
Paired scatterplot demonstrating changes in participant comfort levels pre- and post-intervention. *P* values are based on the Wilcoxon ranked sum test. Y axis represents the Likert scale from 5 = extremely confident to 1 = extremely unconfident. Open circles represent individuals who rated confidence at each interval. 1 circle = 1 individual.

There was a statistically significant change in pediatric residents’ confidence in several areas using a 5-point Likert scale (Fig. 1). Our primary objective was to increase resident confidence in providing nutritional strategies to patients trying to manage weight in an outpatient setting. Residents demonstrated improved confidence in this area (*P* < 0.01). As far as other measures of confidence are concerned, residents improved their confidence to find clinical resources and references on the USDA MyPlate website (*P* < 0.01), refer patients to high-quality digital content to meet their weight loss and fitness goals (*P* < 0.01), and use evidence-based principles and resources to evaluate the safety and efficacy of weight loss programs (*P* < 0.01).

## DISCUSSION

Despite the fact that physician nutrition education has been scrutinized by trainees, governing bodies, and medical schools for many decades, residents and PCPs continue to cite a lack of knowledge and training as instrumental in how frequently they counsel patients with diseases related to overnutrition (2,11). Huang et al (7) noted that physician confidence was one of the major reasons that physicians provide insufficient guidance on weight management strategies (7). The works of Han et al (3), McLeod et al (6), and Vetter et al (5) also support the notion that increased confidence will lead to increased counseling in the outpatient setting. Based on this work, it may not be requisite to increase the knowledge of PCPs to improve outpatient counseling. It may, in fact, only be requisite to increase their confidence in their ability to provide nutritional education. Therefore, we propose that a curriculum designed to have positive effects on resident confidence should also lead to improvement in patient care.

Our residents showed improved confidence in several different measures. These included improved confidence in their use of online resources, referring patients to high-quality digital content to meet their weight loss and fitness goals, and providing counseling. In our institutional needs assessment, residents frequently noted that they felt as though they had few strategies to provide for healthy eating. We focused on giving a variety of strategies, including choosing between 2 comparable products based on sodium content, considering the length of the ingredient list as a surrogate for processing, and checking fiber content as a marker for whole grains. These types of strategies were woven throughout the curriculum, and the residents demonstrated in the confidence data that these strategies led them to have more confidence in their ability to provide counseling.

One of the limitations of our study was that the curriculum was initially prepared in the fall of 2019 and the early portion of 2020. The SARS-CoV-2 pandemic in March of 2020 had significant impacts on our research. First, in-person learning was significantly decreased. Our ability to do in-person lectures, grocery store tours, and even outpatient clinical learning was significantly hampered. Second, while we originally intended for the study to last closer to 18 months to get all residents through the various rotations this did not happen due to adjustments to resident clinical and teaching schedules. Fortunately, since the curriculum was multimodal, we were able to work through these barriers to provide small-group education.

In future studies, we would recommend tracking individual residents to ascertain if residents who complete the curriculum have a statistically significant improvement in knowledge or change in attitude compared with those who do not complete the curriculum. Also, it would be interesting to evaluate each learning activity individually to determine if 1 of the 3 learning activities was more or less influential than the others.

Other limitations may include the fact that there were no assurances that each resident would complete the entire curriculum as some residents may not be available for the lecture or be able to complete the outpatient module. It is also possible that baseline knowledge deficits between the first- and third-year residents influenced the results. However, we did try to separate them by PGY in the data for this reason. Finally, we were unable to find a validated questionnaire. Therefore, one of the limitations of our study is the use of an unvalidated questionnaire.

In summary, we present an interactive, hands-on nutritional curriculum developed that leads to improved confidence among pediatric residents to provide nutritional counseling. This improved confidence is likely to lead to more frequent counseling. Finally, we recommend the application of interactive, mixed-learning models to meet the nutritional educational needs of physician trainees.

## Supplementary Material


