# Supplementary material for: Application of an Interactive, Hands-On Nutritional Curriculum for Pediatric Residents
Source: JPGN Rep. 2023 Nov 13;4(4):e384. doi: 10.1097/PG9.0000000000000384 (PMC10684231; doi:10.1097/PG9.0000000000000384)
Supplement: Supplementary file 2 [file pg9-4-e384-s002.pdf]

Outline for Grocery Store Learning Activity

1. Welcome and Introduction
    a. Objectives
        i. Help participants be able to better counsel patients on healthy eating
            1. Pediatric residents will practice family motivational interviewing and then apply a SMART goal to a clinic family.
        ii. Be able to compare and contrast the nutritional density of foods
            1. Pediatric residents will describe the nutritional density of various foods including compare and contrast the nutritional density of 2 given food choices within the same category. They will also be able to estimate portion sizes of common foods.
            2. Pediatric residents will retrieve the USDA recommendations on the My Plate website to estimate kilocalories, nutritional content of foods, and appropriate portion sizes for patients.
        iii. Understand how to make small changes
            1. Pediatric residents will recognize that small gains represent successful management of disease and applaud the efforts of patients to change.
    b. What we are NOT doing
        i. Trying to make participants into dieticians
        ii. Not talking politics of various products (Ie animal treatment, environmental concerns, etc). We are trying to learn about healthy foods and principles.
        iii. Try to avoid labeling foods as healthy or unhealthy
2. General approaches
    a. Ask the group, “How do you approach nutritional counseling?”
        i. Page 1 of the handout has some thoughts. Give them one minute to consider their answers then discuss as a group.
        ii. Who do participants counsel/What triggers participants?
            1. Often, it is based on BMI
                a. Discuss with the group when the BMI is appropriate
                    i. <2 yo
                    ii. For use in a population. It is not predictive for individual patients
                        1. FYI Weight loss of 1lb/wk is about 300-500kCal
                        2. This is an opportunity to emphasise that small changes can make a significant difference in health
            2. Doctors often counsel patients about nutrition when they are at high risk for obesity related complications

a. What risk factors (RF) listed in the table on page one are actually risk factors for obesity?
b. RF for obesity include:
   i. Maternal Pregravid Weight
   ii. Parental BMI
   iii. Rapid weight gains in infancy
   iv. >2 hrs/day screen time
   v. Intake of Sugar sweetened beverages
   vi. <8 hrs/night sleep
   vii. Single Parent Family
   viii. Participation in WIC
   ix. Parental Restrictive Feeding

iii. How long do you counsel about obesity? When do you do it?
   1. Samples answers may include:
      a. At the end of the visit
      b. <30 sec

3. How is a store set up?
   a. As a business entity stores are set up to make money
      i. Fruits and veggies first help participants feel healthier as participants move throughout
      ii. Flowers help improve shopping experience
      iii. End caps draw shoppers into the aisle
      iv. Participants don't have to shop just the perimeter to be healthy
4. Speaking of which…How do we decide what is a healthy diet anyway?
   a. Mediterranean Diet has the best data behind it
   b. There is no one right way to eat…We want to help participants see simple ways to counsel patients
      i. These simple changes represent small changes and deserve praise
   c. We will be using the *Dietary Guidelines for Americans 2015-2020 edition* to help decide what is a healthy diet
5. Briefly review the new food label on Page 2 of the participant's worksheet
      i. Ask the students to explain "Nutrient Density."
6. The Produce Aisle
   a. There are 5 ways we get our produce. Fresh, Dried, Canned, Frozen, Juiced.
      i. Fresh
         1. To encourage discussion ask the group, "Is there a fruit or vegetable participants have not tried but would like to?" or "Is there a fruit or vegetable participants like but don't eat often?"
            a. Why not?
            b. What barriers do participants or patients face in eating fresh produce?
         2. How can participants overcome barriers to getting more fresh produce?

        a. Preparation
            i. Prepare several at a time
            ii. Pre-Packaged
                1. Look at the pre-packaged items and compare the cost
        b. Time
            i. Consider Steaming vs boiling
                1. Steaming is both faster and improves nutritional density
        c. Flavor
            i. Discussed in section 7.b.i
        d. Cost
        e. Each participant should pick one fruit and one vegetable to evaluate. This can be done from cards provided with nutritional information on it
            i. Have participants fill out the table in their handout

7. The Deli
    a. Ask the class, "What are some tips/tricks participants have for on-the-go healthy eating?"
    b. Consider condiments
        i. What are ways participants can add flavor to participantsr meals that are healthier
            1. Spice mixes, Hummus, Peanut Butter, Ketchup/Mustard vs Mayo, Vinegars
                a. This is another opportunity to emphasize small changes by suggesting interventions such as putting your own condiments on foods ordered at restaurants or swapping higher calorie condiments for lower calorie options
8. The Canned Goods
    a. Per the USDA Cans are the #1 recycled thing in the USA
    b. What is are canned goods stored in?
        i. Water, Juice, light syrup, heavy syrup, and salt
        ii. Water is lower in kCal than 100% Juice which lower than syrups
            1. Compare the caloric content and added sugar in the same fruit of the same brand that has been packaged differently
    c. Sodium content is a great thing to watch particularly in canned vegetables
        i. Avoid the adage, "Lay off the salt shaker."
            1. Most of our sodium is actually added by manufacturers and not at the dinner table
        ii. Rinsing canned goods can decrease the sodium content by as much as 40%
        iii. Compare the same brand of several vegetables in standard packaging vs low sodium
    d. Look at the added sugar content

    i. This is a good marker of processing
    ii. Ask each participant to find the fruit/veggie that they found earlier and compare it in its canned form
  e. Let’s apply this to a well-known canned protein…Peanut Butter
    i. Have each participant select a different type of PB
    ii. As a group compare the nutritional content of the PB using added sugars and sodium content
    iii. This a great place to talk about the length of the ingredients list. The longer the list usually represents more processed foods.
      1. Ingredients are listed by weight
        a. Teaching patients to use ingredient length list is a quick tip that can be shared in clinic
      2. Notice that the PB that have longer ingredients lists also often have more sodium and added sugar
  f. Remember that canned foods have a long shelf life and can therefore decrease waste

9. The Meat Department
  a. Portion size here is more difficult to measure
    i. Provide the participants with a deck of cards to see what 4 oz of meat looks like
  b. What are examples of lean protein?
    i. Fish
      1. The goal is for two servings of fish per week on the Mediterranean diet
      2. Fish cooks quickly
      3. Talapia and Cod are more mild flavored for people who don’t like the taste of fish
        a. There are many “atypical” ways that fish can be prepared as well to improve the taste for some consumers
          i. For example: fish tacos
    ii. >90% Lean
    iii. Loin/Round cuts more likely to be lean
    iv. Poultry tends to be lean
      1. As long as the skin is removed

10. The Bread Aisle
  a. What is the difference between whole wheat and whole grain?
    i. Whole grain does not remove the bran
    ii. Whole wheat is usually white bread that has color/flavor added back in
    iii. Whole grain is typically >3g of fiber per serving
    iv. Have each participant select one whole grain and one whole wheat item.
      1. Compare the weight of the two
      2. Compare the fiber content

            3. Reiterate the previous lessons and compare the sodium and added sugar content
        v. "What are the benefits of a high fiber diet?"
            1. May lower cholesterol (Soluble)
            2. Makes stools more consistent
            3. Decreases rates of colon cancer
            4. Increases satiety
    b. Other grains
        i. Note that other grains like whole wheat pasta or rice take longer to cook.

11. The Cereal Aisle
    a. What does the typical American breakfast look like?
        i. High in sugar, low in fiber/protein
            1. Add lean meats, eggs, etc. to improve satiation with breakfast
    b. Activity
        i. Find 1 Cereal with 3+ g of fiber per serving
        ii. Find 1 Cereal with 1g or less of fiber per serving
        iii. Compare the calories, added sugar, and sodium
        iv. Place these in participant's cart. We will look at them again later

12. The Frozen Goods
    a. Frozen goods are generally frozen w/in 24 hours of peak freshness. This is true for both produce and meats.
    b. About 20% of the fresh fruits and veggies bought in america are discarded
    c. Frozen goods have a much longer shelf life
    d. Have each participant find the fruit and vegetable the previously evaluated then fill out the table
        i. Discuss the findings
    e. Look for pasta/rice/quinoa/breads in the frozen aisle
    f. Allow the participants to create a healthy meal from only frozen foods by filling out the section on the handout
        i. When looking at frozen meals consider meals <400-500 kcal and <600 mg sodium
        ii. Participants could add a fresh fruit/veggie/dairy item to participants meal to beef it up and improve the nutritional density

13. The Dairy Aisle
    a. Why is it that milk is such an important food group?
        i. RDA, Food Pyramid, MyPlate
            1. Note that the USDA came up with all of this
        ii. When choosing dairy it is important to focus on the fat content
            1. How can participants tell a "good fat" from a "bad fat"
                a. Saturated vs unsaturated
                b. Solid vs liquid
                c. Note that the fats in dairy are mostly solid and saturated

            d. You can compare these fats to shortening, vegetable oil, olive oil, etc
        2. Fats are important though
            a. Helps satiation
                i. Dressings on salads help make the meal more satiating
        3. Have the group pull out the cereal and PB from earlier and look at the fat content
            a. Changing the fat content of dairy products would represent another small and simple change

14. The Snack Aisle
    a. Consider the following:
        i. Why do we snack?
            1. Frequently is it because:
                a. People are hungry
                b. People are bored
                c. The foods are readily available or convenient
                    i. People will in fact eat more fruits and veggies if they are prepared
                d. Social pressures
        ii. What snacks are readily available to participants at work/home?
        iii. Apply what we have learned so far today to pick out 1-2 healthy snacks that participants could use at work.
            1. Review what snacks the students brought back.
                a. Samples of healthy snacks
                    i. Nuts/Seeds-Healthy fats and lean protein
                    ii. Whole grain chips
                    iii. Popcorn
                    iv. Rice cakes

15. Summary
    a. Small changes make a big difference
    b. Participants can teach a patient to select foods with increased fiber, decreased sodium, decreased added sugar, etc. in < 30 seconds of clinic time
        i. These things can make major dietary impacts
    c. Goals
        i. Briefly discuss SMART goals
            1. Specific
            2. Measurable
            3. Achievable
            4. Realistic
            5. Timing
        ii. Have the patients consider their most recent nutritional counseling experience. Write 1-2 SMART goals for that patient.

16. Resources

    a. Vos MB, Welsh J. Childhood obesity: update on predisposing factors and prevention strategies. *Curr Gastroenterol Rep*. 2010;12(4):280-287. doi:10.1007/s11894-010-0116-1
    b. Dev DA, McBride BA, Fiese BH, Jones BL, Cho H; Behalf Of The Strong Kids Research Team. Risk factors for overweight/obesity in preschool children: an ecological approach. *Child Obes*. 2013;9(5):399-408. doi:10.1089/chi.2012.0150
    c. (2020, September 11, 2018). "Childhood Overweight and Obesity." Retrieved May 2020, 2020, from https://www.cdc.gov/obesity/childhood/index.html.
    d. U.S. Department of Health and Human Services and U.S. Department of Agriculture. *2015 – 2020 Dietary Guidelines for Americans*. 8th Edition. December 2015. Available at https://health.gov/our-work/food-and-nutrition/2015-2020-dietary-guidelines/
    e. Steinbach, S. (2018). Grocery Store Tour Educational Guide. United Dairy Industries of Michigan.

17. Preparation
    a. Photos of DGA, My Plate, Food Pyramid
    b. New food label
    c. Pens
    d. Participant worksheet
    e. Fruit and vegetable cutouts
    f. A Deck of Cards
    g. Meat Cutouts
