# Supplementary material for: Application of an Interactive, Hands-On Nutritional Curriculum for Pediatric Residents
Source: JPGN Rep. 2023 Nov 13;4(4):e384. doi: 10.1097/PG9.0000000000000384 (PMC10684231; doi:10.1097/PG9.0000000000000384)
Supplement: Supplementary file 3 [file pg9-4-e384-s003.pdf]

Grocery Store Worksheet

What is your approach to dietary and/or nutritional counseling? Consider the following:

Who do you counsel?

What triggers you to recommend lifestyle changes?

What changes do you focus on?

When does this counseling take place during the visit?

How much of the visit do you focus on counseling?

Do you find your counseling important and/or effective? Why or Why not?

Circle the risk factors for obesity.

Maternal Pregravid Weight	Maternal GTT	Parental BMI	Rapid Weight Gains in Infancy
Infant Birth Weight	>2 hours/day Screen Time	Breastfeeding	Socio-Economic Status
Intake of Sugar Sweetened Beverages	<8 hrs of Sleep per Night	Two Parent Family	Parental Knowledge of Nutrition Labels
Attendance at Child Care	Participation in Head Start	Parental Restrictive Feeding	Maternal Smoking
Fat Content of Milk	Single Parent Family	Sugar Added to Baby Formula at <1 yo	Participation in WIC

Is there a fruit or vegetable you have not tried but would like to? Is there a fruit or vegetable you like but don't eat often? Why not? What barriers do your patients face in eating fresh produce?

Fruit ____________	Cost	Portion Size	kCal	Sodium Content	Added Sugars	Micronutrients	Overall Nutritional Value
Fresh							
Frozen							
Canned							
Other							

Vegetable ____________	Cost	Portion Size	kCal	Sodium Content	Added Sugars	Micronutrients	Overall Nutritional Value
Fresh							
Frozen							
Canned							
Other							

Please create 1-2 healthy meals using only frozen foods. Please make two separate meals (IE breakfast and dinner, lunch and dinner). Do not repeat an item you found.

First Meal: Breakfast/Lunch/Dinner

1.
2.
3.
4.
5.

Second Meal: Breakfast/Lunch/Dinner

1.
2.
3.
4.
5.

Why do we snack? Make a list of snacks you usually see at work. Next, find 1-2 healthier snacks that you could take with you to work.

**Snacks at Work**	**“Better” Snacks**

List 3-5 Takeaway points that you can use in your next patient encounter

1.

2.

3.

4.

5.
