# Supplementary material for: Application of an Interactive, Hands-On Nutritional Curriculum for Pediatric Residents
Source: JPGN Rep. 2023 Nov 13;4(4):e384. doi: 10.1097/PG9.0000000000000384 (PMC10684231; doi:10.1097/PG9.0000000000000384)
Supplement: Supplementary file 4 [file pg9-4-e384-s004.pdf]

Grocery Store Worksheet

What is your approach to dietary and/or nutritional counseling? Consider the following:

Who do you counsel?

What triggers you to recommend lifestyle changes?

What changes do you focus on?

When does this counseling take place during the visit?

How much of the visit do you focus on counseling?

Do you find your counseling important and/or effective? Why or Why not?

Circle the risk factors for obesity.

**Maternal Pregravid Weight**	Maternal GTT	**Parental BMI**	**Rapid Weight Gains in Infancy**
Infant Birth Weight	**>2 hours/day Screen Time**	Breastfeeding	Socio-Economic Status
**Intake of Sugar Sweetened Beverages**	**<8 hrs of Sleep per Night**	**Two Parent Family**	Parental Knowledge of Nutrition Labels
Attendance at Child Care	Participation in Head Start	**Parental Restrictive Feeding**	Maternal Smoking
Fat Content of Milk	**Single Parent Family**	Sugar Added to Baby Formula at <1 yo	**Participation in WIC**

Is there a fruit or vegetable you have not tried but would like to? Is there a fruit or vegetable you like but don't eat often? Why not? What barriers do your patients face in eating fresh produce?

Fruit ____________	Cost	Portion Size	Sodium Content	Added Sugars	Micronutrients	Overall Nutritional Value
Fresh						
Frozen						
Canned						
Other						
Vegetable ____________	Cost	Portion Size	Sodium Content	Added Sugars	Micronutrients	Overall Nutritional Value
Fresh						
Frozen						
Canned						
Other						

Please create 1-2 healthy meals using only frozen foods. Please make two separate meals (IE breakfast and dinner, lunch and dinner). Do not repeat an item you found.

First Meal: Breakfast/Lunch/Dinner

1.

2.

3.

4.

5.

Second Meal: Breakfast/Lunch/Dinner

1.

2.

3.

4.

5.

Why do we snack? Make a list of snacks you usually see at work. Next, find 1-2 healthier snacks that you could take with you to work.

**Snacks at Work**	**“Better” Snacks**

List 3-5 Takeaway points that you can use in your next patient encounter

1.

2.

3.

4.

5.
