# Supplementary material for: Application of an Interactive, Hands-On Nutritional Curriculum for Pediatric Residents
Source: JPGN Rep. 2023 Nov 13;4(4):e384. doi: 10.1097/PG9.0000000000000384 (PMC10684231; doi:10.1097/PG9.0000000000000384)
Supplement: Supplementary file 5 [file pg9-4-e384-s005.pdf]

Today we will be exploring several online and mobile based resources that you can use in an outpatient setting either for personal reference or as materials for you patients. To do this we will be using a sample case. Notably, each website has lots of other resources that we will not be able to address during this activity. Please explore the resources for other tools that might be of benefit to you.

Maya is a 14 yo F. She weighs 187lbs and is 63" tall. She plays 45 min of basketball each day.

1-How many calories does Maya need in a day?

A. First, go to choosemyplate.gov

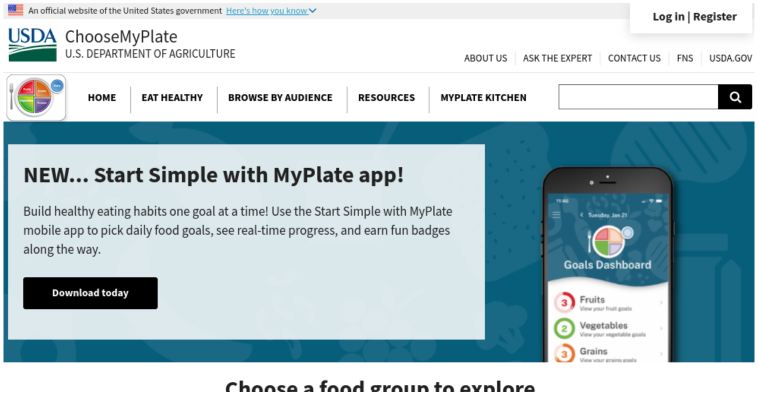

B. Scroll down and select "Get Your My Plate Plan."

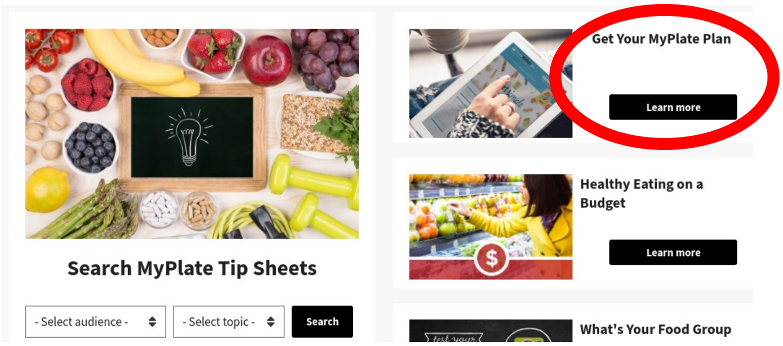

C. Click start and enter Maya's information.

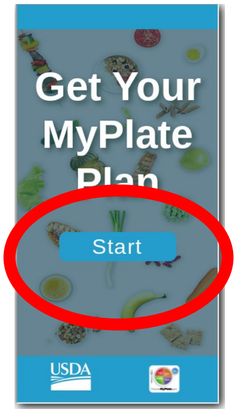

D. Maya needs ___________kCals

2-How many servings of fruits and vegetables does Maya need in a day?

A. Scroll to the bottom of the page. Select her 2000 kCal plan for 14+

Age Group	Calorie Level								
Ages 2-3	1000	1200	1400						
Ages 4-8	1200	1400	1600	1800	2000				
Ages 9-13	1600	1800	2000	2200	2400	2600	2800	3000	3200
Ages 14+	1600	1800	2000	2200	2400	2600	2800	3000	3200

B. Maya needs _______________ servings of fruit and ________________ servings of vegetables

3. Maya has been reading about supplements for athletes. A lot of her friends are touting "beta-alanine" produced by LiveLong. She wants to know if it is effective. Her mom wants to know if it is safe.

A. Go to the UM Clinical Homepage. At the top select "clinical references."

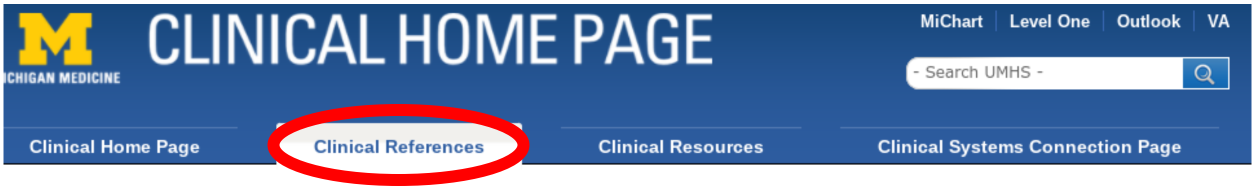

B. Under "Pharmacy Resources" select "Natural Medicines."

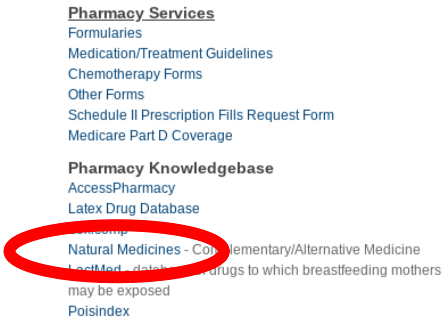

C. In the search bar type "beta-alanine" and select the LiveLong brand. Is this safe? Is it effective?

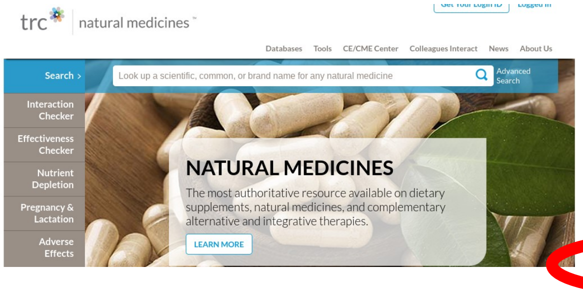

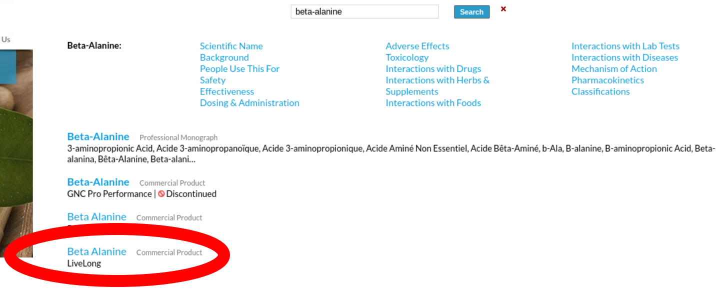

4. Maya's mother notes that basketball season is just wrapping up and says, "Maya walks her dog around the block day. I think she should get out more." How much physical activity should a teen get in a day?

A. Go to health.gov and click on "Physical Activity."

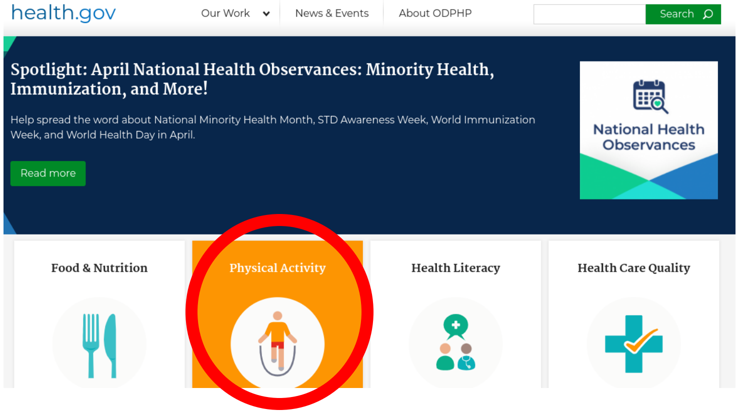

B. On the left-hand side of the screen select "Current guidelines" and then "Download the complete second edition physical activity guidelines." These are evidence-based guidelines from HHS and the American College of Sports Medicine.

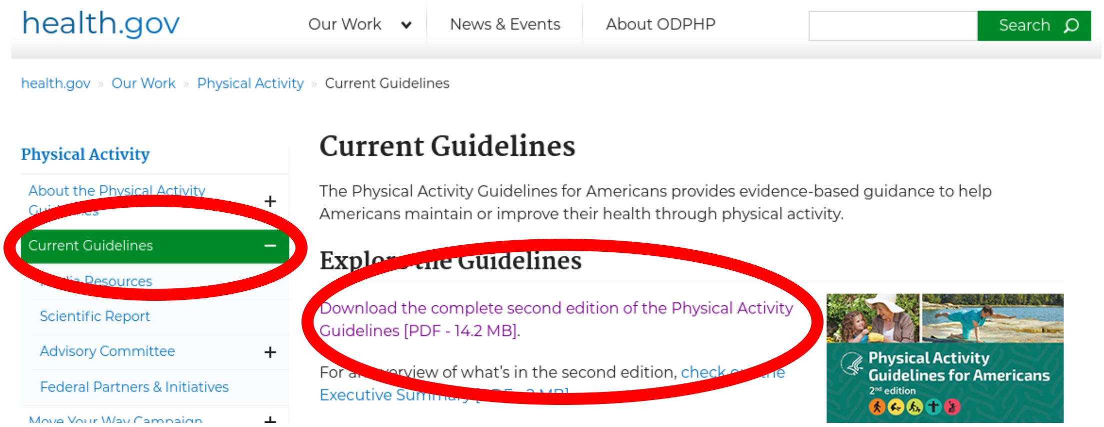

C. Scroll down to Chapter 3 on children and adolescents. Look at the "Key Guidelines for School Aged Children and Adolescents" on page 48. How much physical activity should Maya get?

D. Maya insists that she does more than walk the dog. What activities qualify as moderate or vigorous? See the tables on page 51 and 52.

5. Maya agrees to monitor her caloric intake during the summer. You commend her on making a healthy choice. She asks for an app that might be helpful in her goals. You recommend MyFitnessPal.

A. On your phone search for the MyFitnessPal app. (You may need to enter your demographic info if you have never used it before)

B. Once in the app select the plus sign at the bottom

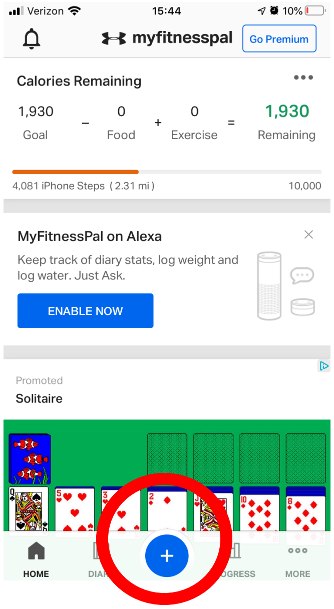

C. Then select add food.

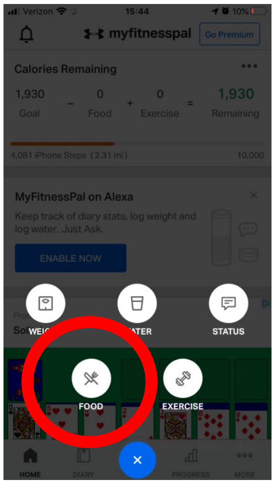

D. Choose a meal when prompted. Then click on the barcode scanner in the right upper corner.

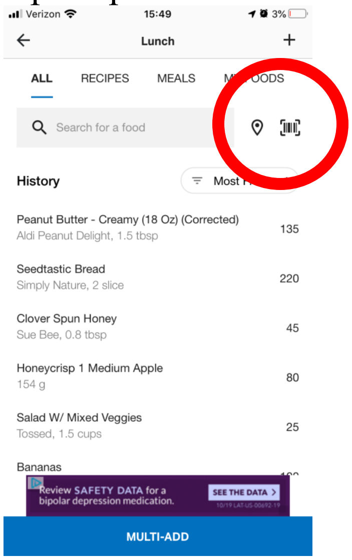

E. Notice that you can look not only at kCals but also micronutrient content of foods. This can track you foods throughout the day.

Maya decides to participate in a summer pledge-drive for the girls basketball team. They are going to have a marathon basketball game with pledges for every hour they play. The event will take place outdoors all day Saturday. She drinks water before and during her event to maintain hydration. However, she faints about 3 hours into the marathon game. Her mother takes her to the ED. You make specific recommendations for days that she plans to participate in vigorous activity for >1hr. Of the following the most appropriate advice for these days is to ingest…

1. Caffeine-containing drinks during and after long activities
2.
3. Carbohydrate drinks before, during, and after long activities
4.
5. Daily amino acid supplements
6.
7. High protein meals 3-6 hours before long activities
8.
9. Increased amounts of water after the activity
10.

Answer: B
Optimal exercise requires that you pay attention to fluids intake before, during, and after activity. Fluid requirements vary by the type of sport, duration of activity, ambient temperature, genetics, body weight, etc. In addition to water, electrolyte and carbohydrate intake becomes important when sports participation lasts longer than 1 hour. Therefore, Maya needs some carbohydrate containing beverages. Notably, excessive water intake puts the athlete at risk for hyponatremia. No evidence supports the need for protein >0.85 g/kg/d in adolescents undertaking aerobic or anaerobic activity. Supplements of amino acids and other proteins have not been show to be helpful in anaerobic activity. High carbohydrate low fat meals have been shown beneficial prior to endurance activities. Energy drinks may contain vitamins and other herbal supplements. These are typically high in calories and low in salts. Both sugars and salts in commercial sports drinks are important but be cautious as many of these products have very high levels of sugar. Because of this many companies have now introduced products that have less sugar. One example is Gatorate's G2 which has 8g of carbohydrates compared to the original which has 22g of carbs.
Adapted from PREP 2011
