# Supplementary material for: Application of an Interactive, Hands-On Nutritional Curriculum for Pediatric Residents
Source: JPGN Rep. 2023 Nov 13;4(4):e384. doi: 10.1097/PG9.0000000000000384 (PMC10684231; doi:10.1097/PG9.0000000000000384)
Supplement: Supplementary file 6 [file pg9-4-e384-s006.pdf]

IP Lecture

1. Objectives
2. Session preparation
    a. Many pediatric residency programs have a history of providing the residents lunch and/or breakfast in conjunction with educational sessions. We attempted to leverage this situation in our pediatric residency program.
    b. We provided snacks prior to the activity. If being used in another setting this meal could represent something typical for that environment. This may include fruits, vegetables, meats, grains, desserts, and/or beverages. Example meals may include (but are not limited to):
        i. A pasta served with salad and bread as a side. Dessert available
        ii. Taco or baked potato bar with meats, beans, rice, cheese, lettuce, tomatoes etc. Beverages available
        iii. Soup, salad, and sandwich with chips and cookies.
    c. We provided food to different team rooms. Alternatively, the food tables could divided into 2 lines with the food arranged differently. We did the following:
        i. One line had the salad placed as the first food available. The second line should had it placed last.
        ii. One line had fruit or vegetables prepared (For ex apples slices). The second table should have whole fruits/vegetables.
        iii. Each table had several large and small plates for participants to choose from. Most participants will choose a larger plate when given a choice so these were consciously limited in number.
3. Who is in control?
    a. What can we learn
        i. Look at the tables (or have participants compare their plates) notice that based upon how the environment was structured will depend on what foods were consumed
            1. In most instances the table with salad first will have more salad missing than the table with salad second
            2. In most instances the table with pre-sliced fruits and vegetables will have more missing from the table than those with whole fruits and vegetables
            3. In most instances participants who chose (or were required) to use smaller plates will have chosen smaller portion sizes
        ii. The point of this activity is to demonstrate that eating healthy is actually more about the environment that we create than it is our own willpower
            1. Play “Hidden Brain” podcast clip
        iii. When it comes to healthy eating you need to be in control. There are lots of ways to do this:

            1. Make ½ your plate fruits and vegetables
            2. Use a smaller plate
            3. Don't clear your plate
            4. Don't let the workers add fats and salts
            5. Ask a restaurant to put ½ your food in a to go container before bringing it

4. Reconsider your breakfast
    a. Note that in the morning most people have a blood sugar in the 80's
        i. The average american breakfast (high in processed sugars) will lead to a blood sugar spike
        ii. Blood sugar will typically bottom out around 60 at 10-11am
            1. So now you are hungrier than you were to start the day
    b. Encourage participants to pick a food for breakfast that will help them feel full
        i. Protein or whole grain
            1. Fruit, Eggs, PB, Protein bars, meat
    c. Don't skip breakfast
        i. People who eat breakfast eat more kCal but have lower BMIs
5. Snacks
    a. People will eat what is available. So make sure there are readily available healthy options
        i. Consider low fat dairy products
        ii. Many people complain about time to prepare food. When cooking cut up an extra apple, banana, carrot, celery, etc for your snacks
    b. Measure out an amount
        i. Don't take the whole bag to the couch
        ii. Have several volunteers select an estimated portion size of various foods. Then provide a properly measured sample. In our sample these were provided visually. Examples of may include:
            1. 2 TBS PB
            2. ¼ c peanuts
            3. 3 C popcorn
            4. ½ C Ice Cream
            5. 1 C Milk
            6. 1 C Cereal
            7. 3 oz meat
            8. 4 oz chicken
                a. Participants should recognize that familiarity with portion sizes in important. Many people will underestimate portion sizes

c. Carry individual snacks instead of relying on what is laying around the office. Again, consider what will make/keep you full

6. Dinner Time
    a. My plate website has lots of goof resources. Bring flyers as examples of ways to eat heathier.
    b. A rotating classroom model can be used to have groups discuss the following. Each table should have flyers from MyPlate.
        i. Group 1-Using *5 Ways* strategy come up with 5 ways to eat a food not presented in the flyers (For example Carrots)
            1. 5 Ways recipes
        ii. Group 2-*MyPlate MyWins*. Look at the flyers provided and select 1-2 things you think that residents struggle with. How could you use the tips provided to overcome these barriers
        iii. Group 3-*10 tips*. Look at the flyers provided and select 1-2 things you think that residents struggle with. How could you use the tips provided to overcome these barriers
7. Activity
    a. It all adds up. There are lots of ways to improve or monitor activity
        i. Pay attention to the pedometer already in most mobile phones
            1. Just recording steps/kCal leads to healthier choices
                a. Do a weekly check-in
                b. Weight, steps, kCals, etc
                c. Use a printer farther away
                d. Walk 2 flights of stairs
                e. Stand for every phone call
        ii. 7 min workouts
            1. These can be done anywhere so let's do part of one together
        iii. Consider home gym equipment
8. Teamwork
    a. Healthy outcomes are improved with others
        i. Get a resident buddy to do things with
        ii. Don't judge each other
    b. Set team goals ideas may include:
        i. Healthy snack of the day/week
        ii. Team stairs
        iii. Water goals-We have residents try to drink the same fluid goal as a given patient on their team
        iv. Pedometer competition-Which resident can get the most steps during the day
9. Goal setting

a. Remember to take health one day and one small goal at a time
b. Recognize your success
c. *Rate Your Plate-Eastern NC* handout

10. References

a. USDA (2020). Retrieved April 2020, 2020, from https://www.choosemyplate.gov/.
b. U.S. Department of Health and Human Services and U.S. Department of Agriculture. *2015 – 2020 Dietary Guidelines for Americans*. 8th Edition. December 2015. Available at https://health.gov/our-work/food-and-nutrition/2015-2020-dietary-guidelines/
c. Vedantam, Shankar, *Creatures of Habit,* https://hiddenbrain.org/podcast/creatures-of-habit, Accessed December 2020
d. Obesity counseling experience for third year medical students. MedEdPORTAL Publications. August 14, 2014. https://www.mededportal.org/publication/9872.
