# Supplementary material for: Application of an Interactive, Hands-On Nutritional Curriculum for Pediatric Residents
Source: JPGN Rep. 2023 Nov 13;4(4):e384. doi: 10.1097/PG9.0000000000000384 (PMC10684231; doi:10.1097/PG9.0000000000000384)
Supplement: Supplementary file 7 [file pg9-4-e384-s007.pdf]

Portions quiz

How Well Do You Know Your Portions?

To test the software please enter your name

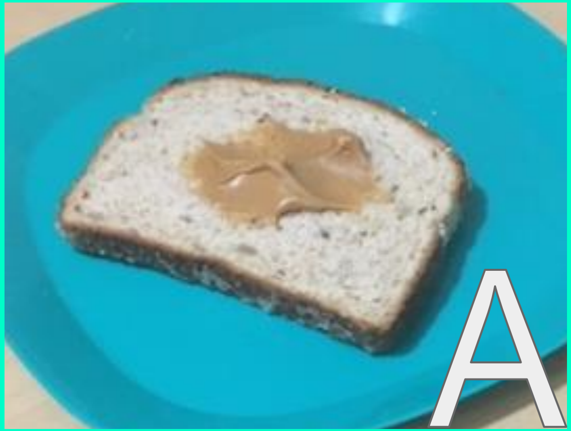

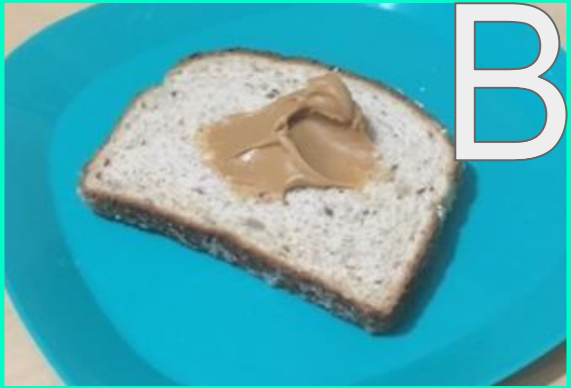

Which of the following is…
2 tbs PB?

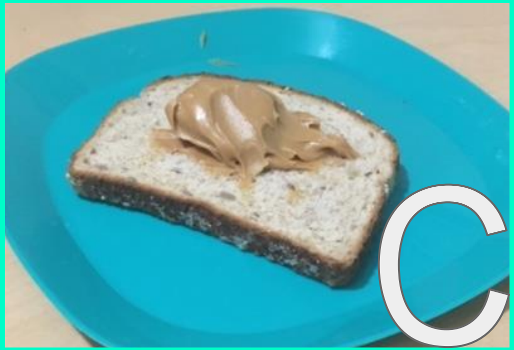

Which of the following is... 2 tbs PB?

A

B

C

D

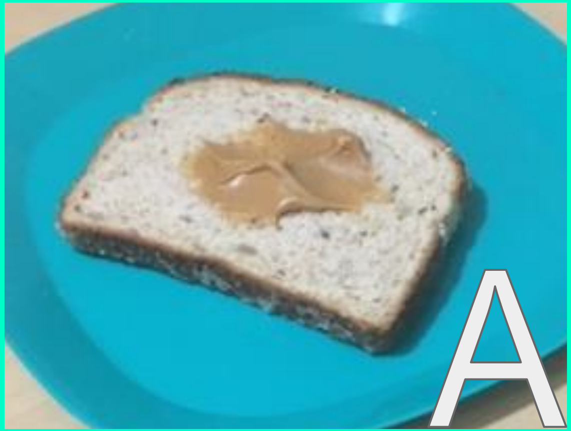

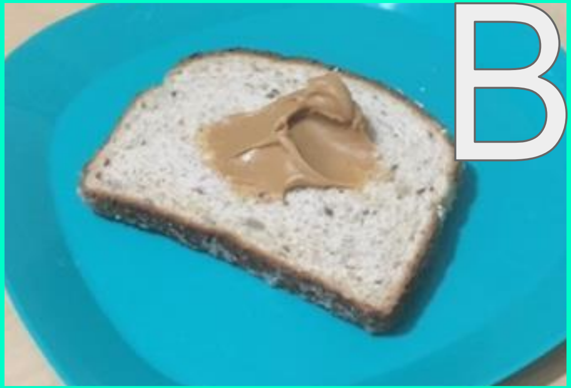

Which of the following is... 2 tbs PB?

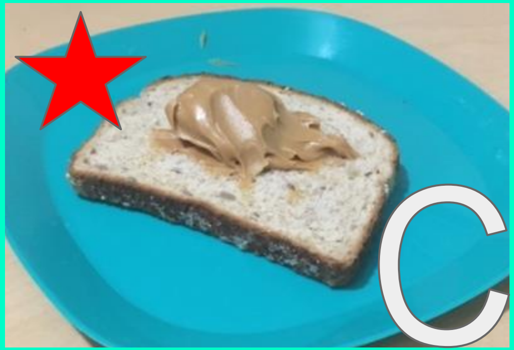

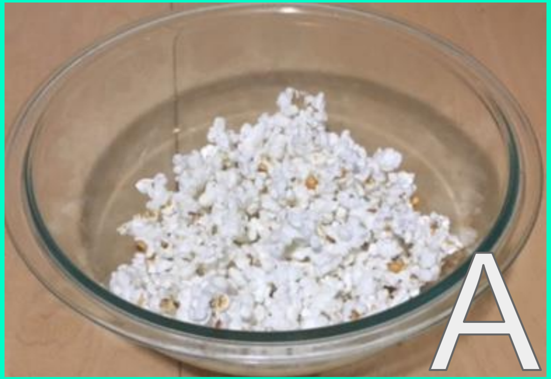

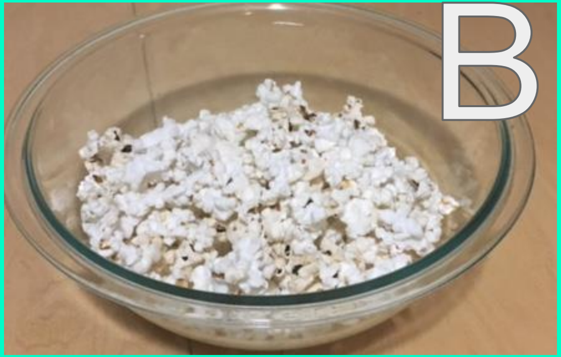

Which of the following is…
3 C popcorn

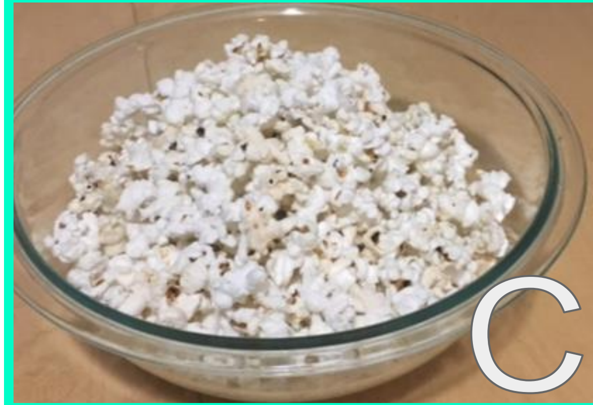

When poll is active, respond at **PollEv.com/coryjones201**

Text **CORYJONES201** to **37607** once to join

Which of the following is... 3 C popcorn

A

B

C

D

Start the presentation to see live content. For screen share software, share the entire screen. Get help at **pollev.com/app**

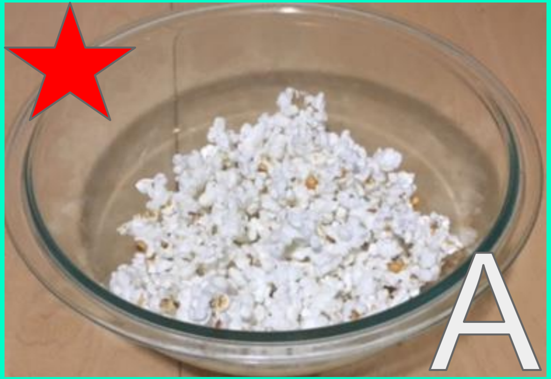

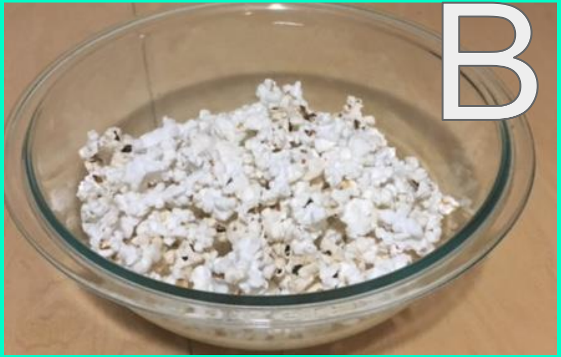

Which of the following is…
3 C popcorn

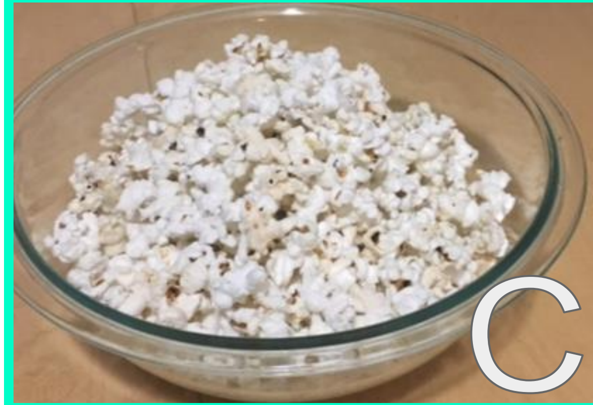

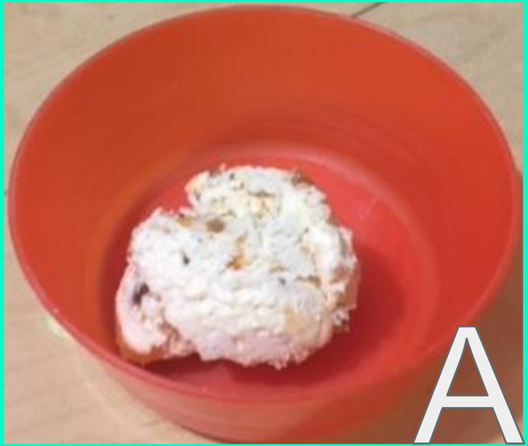

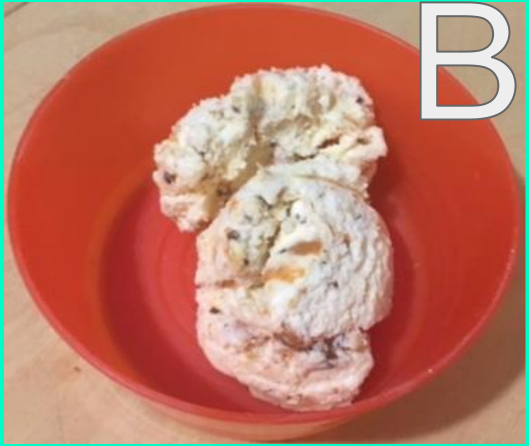

Which of the following is… ½ C ice cream

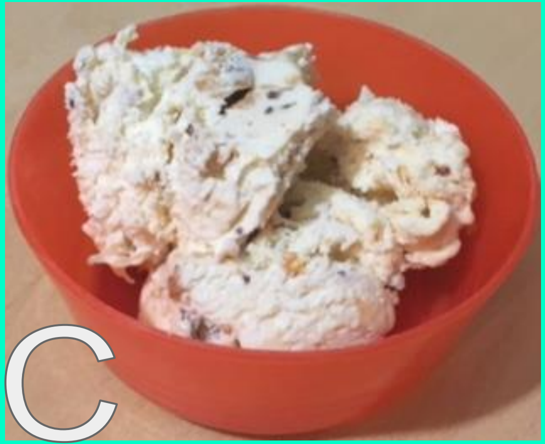

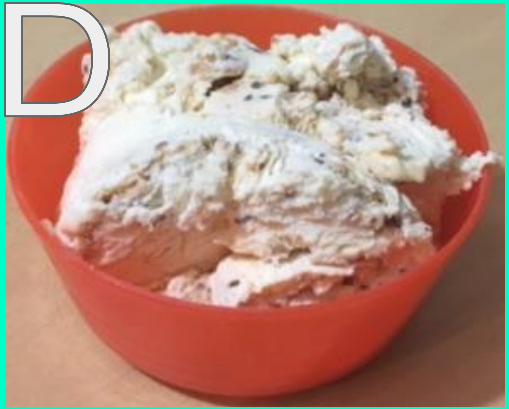

When poll is active, respond at **PollEv.com/coryjones201**

Text **CORYJONES201** to **37607** once to join

Which of the following is… ½ c ice cream

A

B

C

D

Start the presentation to see live content. For screen share software, share the entire screen. Get help at **pollev.com/app**

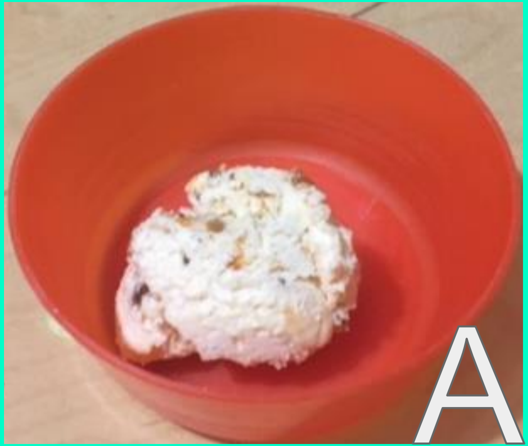

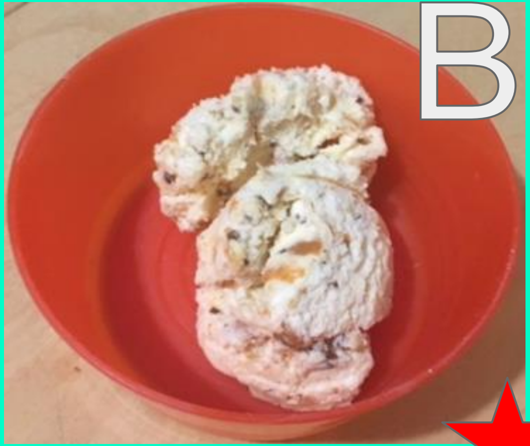

Which of the following is...
½ c ice cream

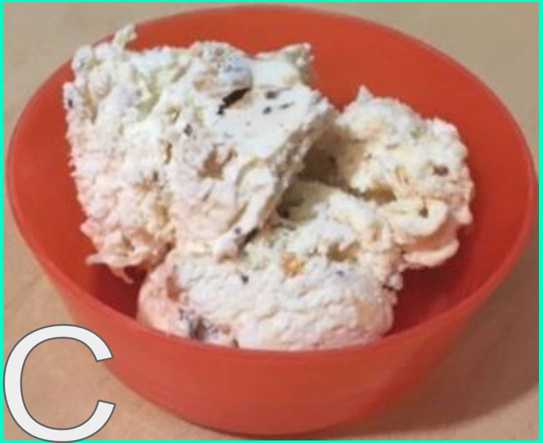

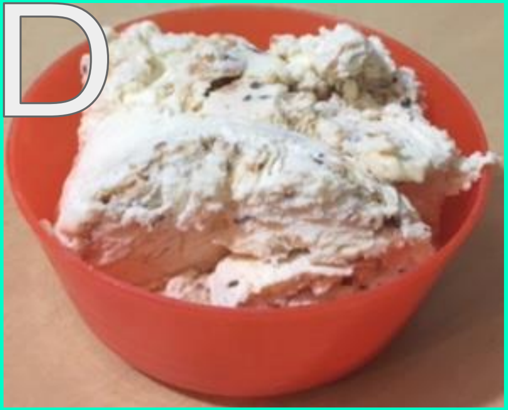

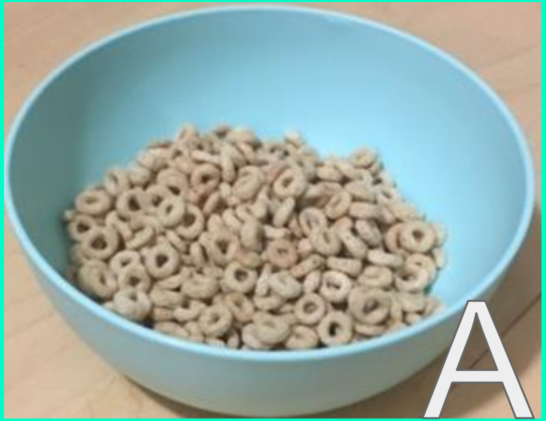

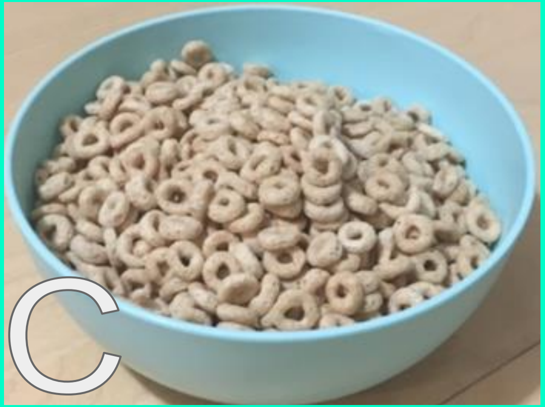

Which of the following is... 1 c cereal

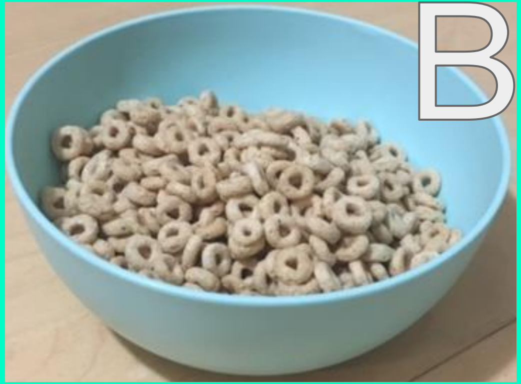

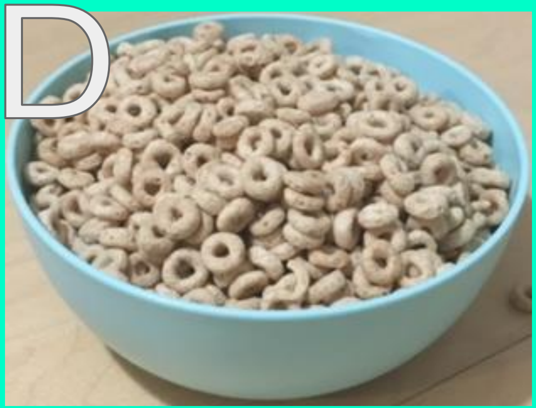

Which of the following is… 1 c cereal

A

B

C

D

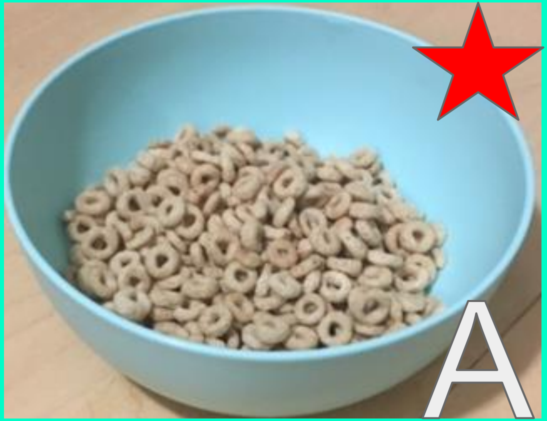

Which of the following is... 1 c cereal

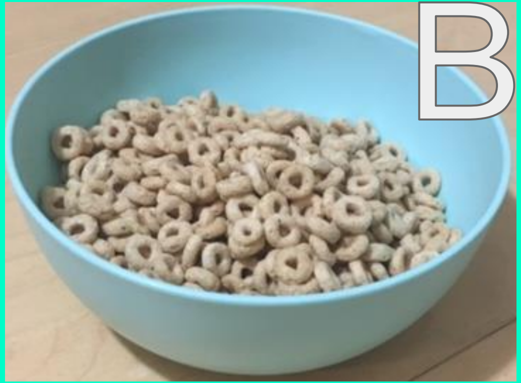

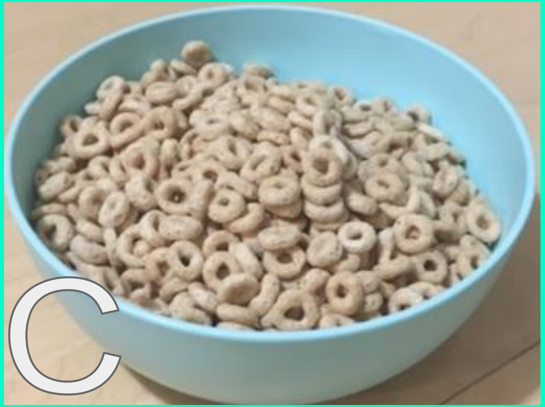

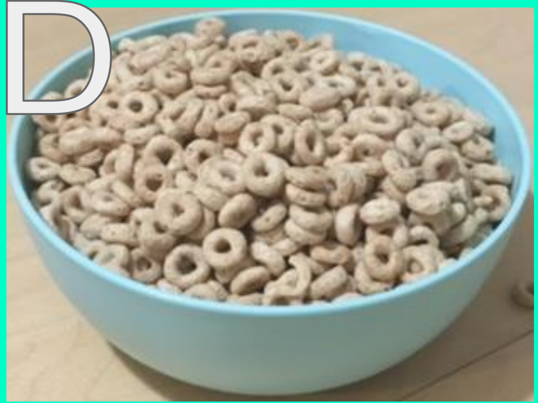

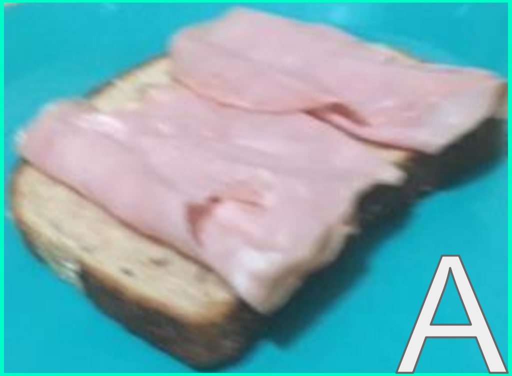

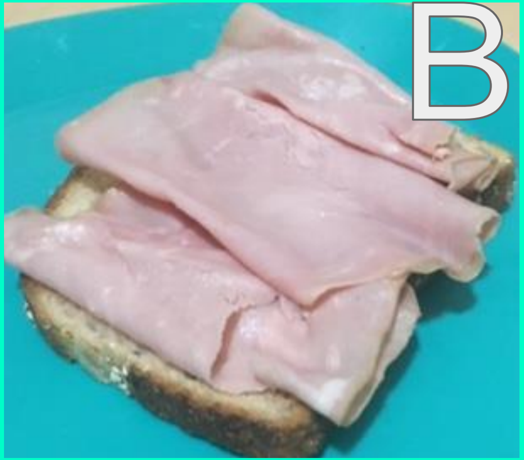

Which of the following is...
3 oz lunchmeat

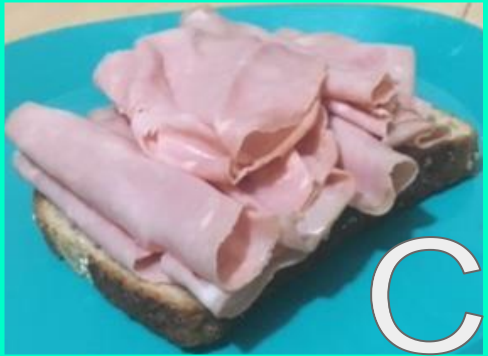

Which of the following is... 3 oz lunchmeat

A

B

C

D

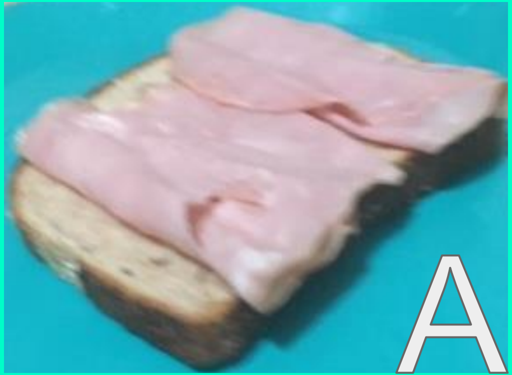

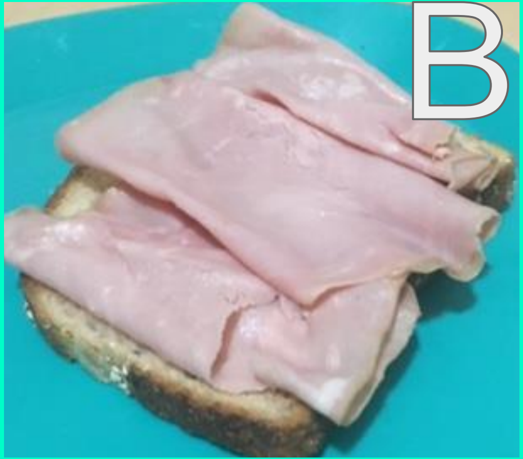

Which of the following is...
3 oz lunchmeat

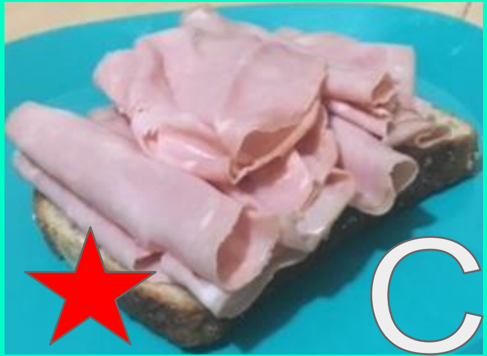

Which of the following is… 1 oz cheese

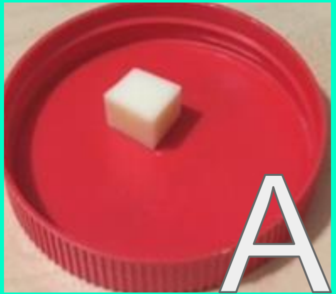

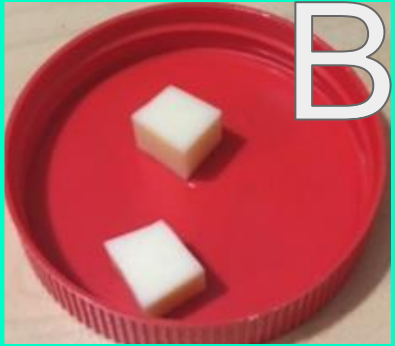

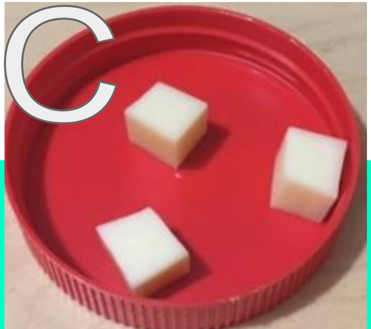

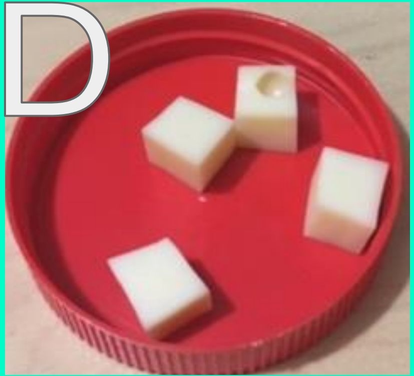

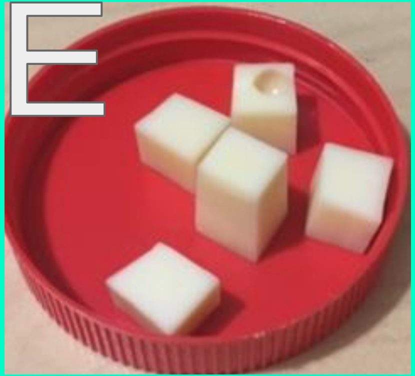

Which of the following is… 1 oz cheese

A

B

C

D

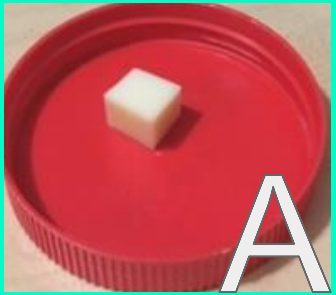

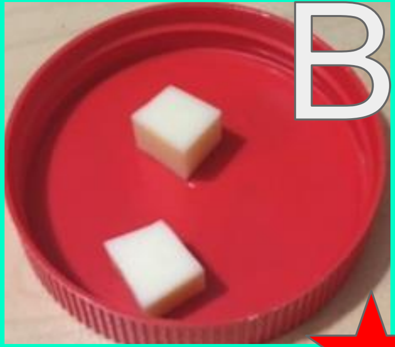

Which of the following is... 1 oz cheese

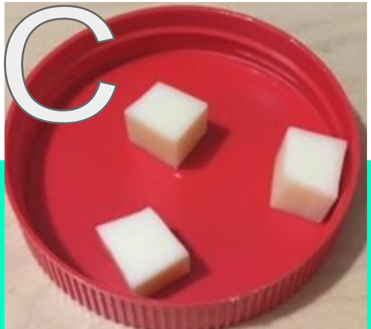

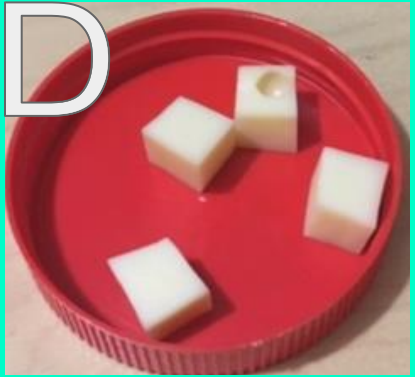

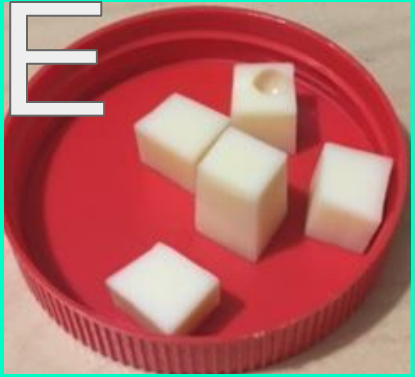

Come up with several ways you could incorporate pumpkin into a toddler diet. Use "5 Ways" as a template.

Start the presentation to see live content. For screen share software, share the entire screen. Get help at **pollev.com/app**

Come up with several ways you could incorporate brown rice into a toddler diet. Use "5 Ways" as a template.

Start the presentation to see live content. For screen share software, share the entire screen. Get help at **pollev.com/app**

You are in charge of designing a MyPlate MyWins infographic entiltled "Meal Planning for One." Name one tip or trick for eating healthy in this situation.

Start the presentation to see live content. For screen share software, share the entire screen. Get help at **pollev.com/app**

You are in charge of designing a MyPlate MyWins infographic entiltled "Prep for Potlucks and Parties." Name one tip or trick for eating healthy in this situation.

Start the presentation to see live content. For screen share software, share the entire screen. Get help at **pollev.com/app**
