# Supplementary material for: Application of an Interactive, Hands-On Nutritional Curriculum for Pediatric Residents
Source: JPGN Rep. 2023 Nov 13;4(4):e384. doi: 10.1097/PG9.0000000000000384 (PMC10684231; doi:10.1097/PG9.0000000000000384)
Supplement: Supplementary file 8 [file pg9-4-e384-s008.pdf]

RATE YOUR PLATE
FOR HEART & BLOOD PRESSURE HEALTH

For each food group, circle the phrase that best describes the way you eat in a typical week. Then write the number of points for that choice in the "points" column. Add up your total points.

Food Group:	Column 1: 1 point	Column 2: 2 points	Column 3: 3 points	Points
**MEAT, FISH & POULTRY, LEGUMES, SEEDS, NUTS**				
◆ Meats such as beef, pork, lamb, veal, lunch meats	Usually eat: high-fat meats such as hot dogs, sausage, ribs, bologna, or regular hamburger	Usually eat: Lean cuts such as pork (loin, leg); veal (most cuts); and beef (round, sirloin, extra lean hamburger), reduced fat hot dogs & lunch meat	Always eat: Lean cuts or rarely eat meat or am a vegetarian	________
◆ Organ meats such as	Usually eat:	Usually eat:	Rarely or never eat	

WHILE YOU WAIT TO START FILL OUT THE WORKSHEET THAT WAS EMAILED THIS MORNING

HEALTHY EATING FOR RESIDENTS

Cory Jones MD

12/2/2020

OBJECTIVES

- Pediatric residents will Identify risk factors for obesity.
- Pediatric residents will understand the advantages and limitations of using BMI to diagnose obesity. They will identify the patient populations for which obesity is an inappropriate measure.
- Pediatric residents will understand how to critically appraise the safety and efficacy of weight loss programs, dietary supplements, and other nutritional products such as meal replacement products.
- Pediatric residents will describe the nutritional density of various foods including compare and contrast the nutritional density of 2 given food choices within the same category. They will also be able to estimate portion sizes of common foods.
- Pediatric residents will be able to list the recommended physical activity guidelines of ACSM. They will use these recommendations to regularly counsel patients on physical activity including that patients get proper sleep, 60 minutes of physical activity a day, and less than 2 hours of screen time.
- Pediatric residents will recognize that small gains represent successful management of disease and applaud the efforts of patients to change.

INTRODUCTION

PRE-COVID

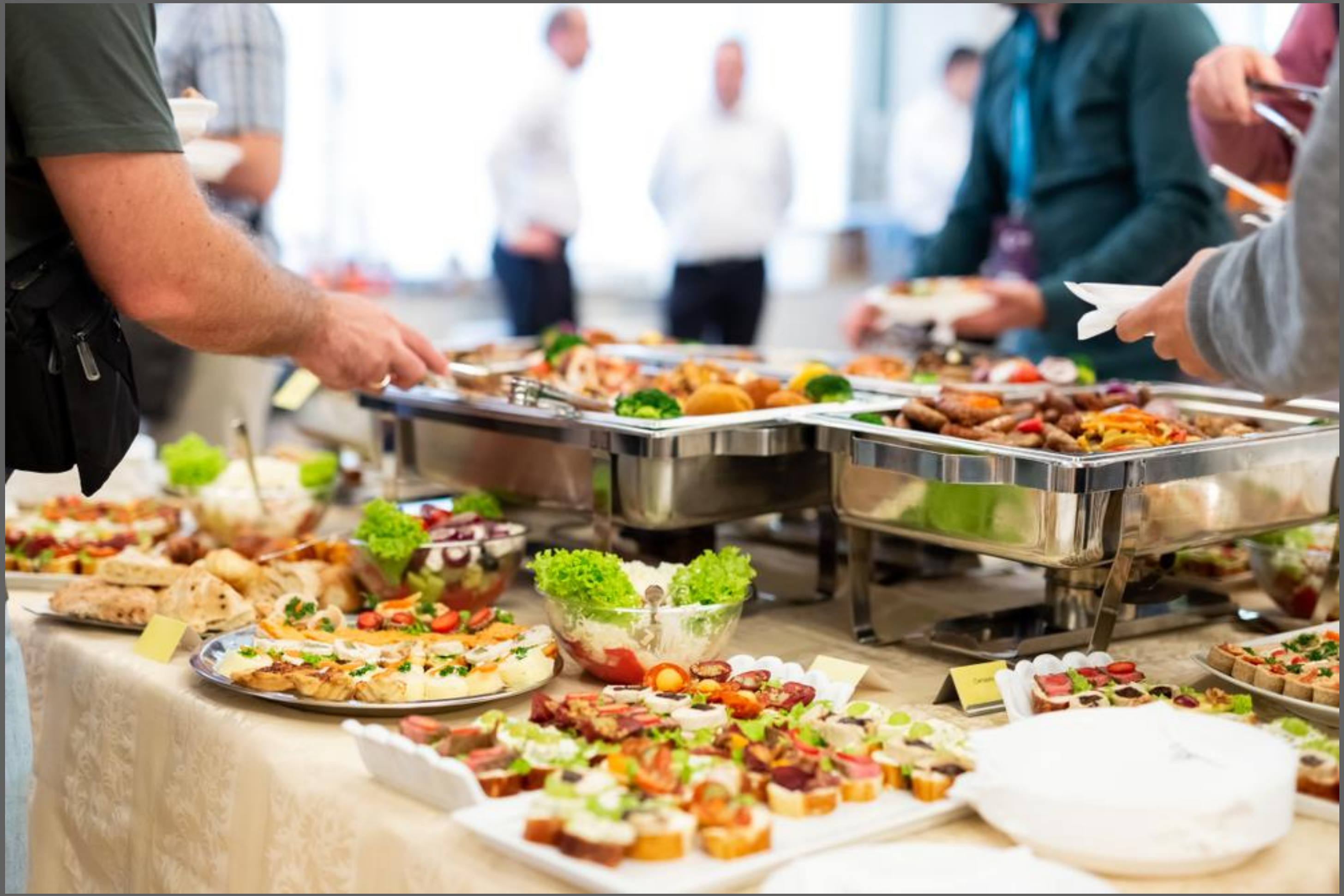

COVID

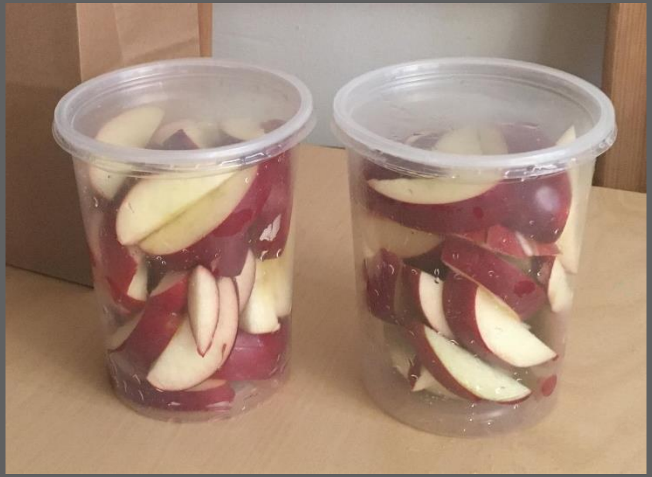

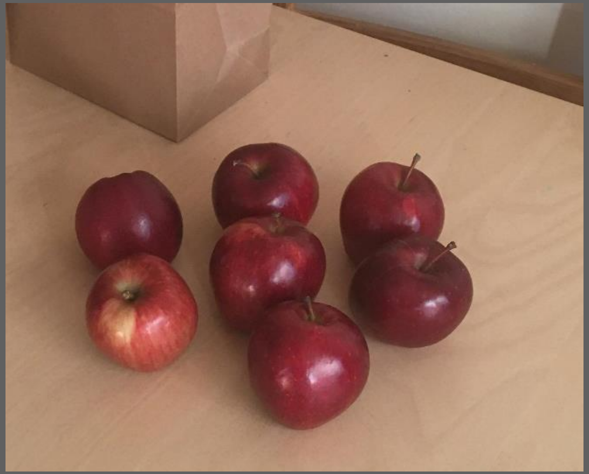

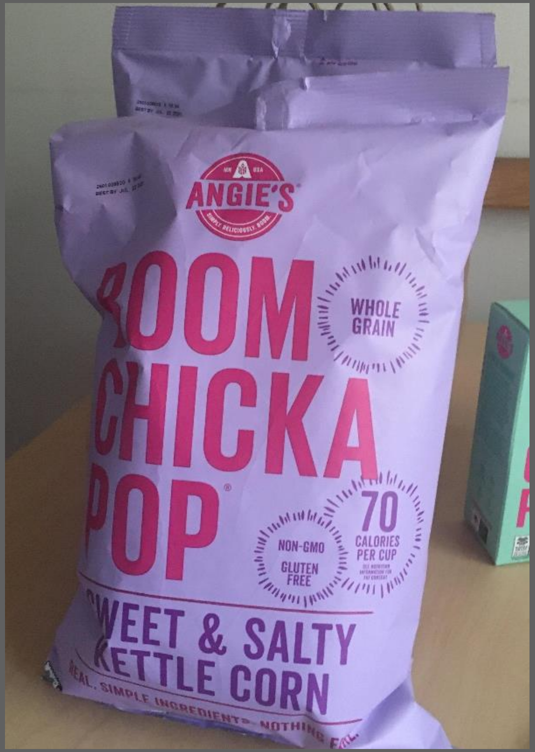
ANGIE'S
CHICKA
POP
WHOLE GRAIN
70 CALORIES PER CUP
NON-GMO
GLUTEN FREE
KETTLE CORN

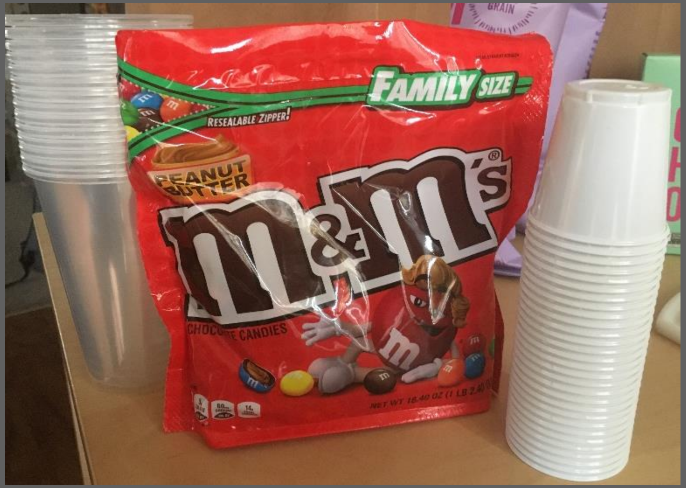
FAMILY SIZE
RESEALABLE ZIPPER!
PEANUT BUTTER
m&m's

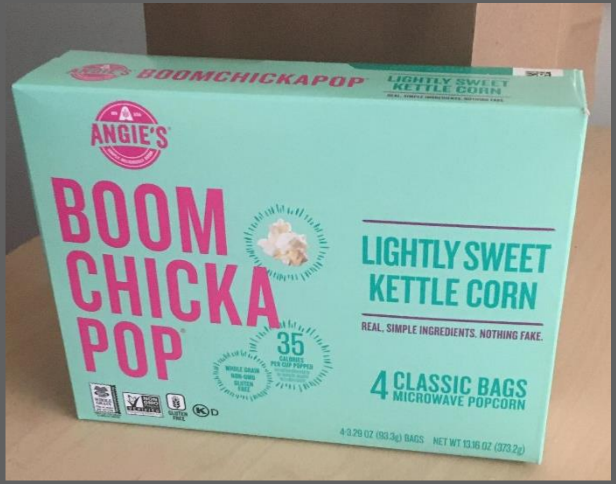
BOOMCHICKAPOP
ANGIE'S
BOOM CHICKA POP
LIGHTLY SWEET KETTLE CORN
REAL. SIMPLE INGREDIENTS. NOTHING FAKE.
35
4 CLASSIC BAGS MICROWAVE POPCORN

APPLES

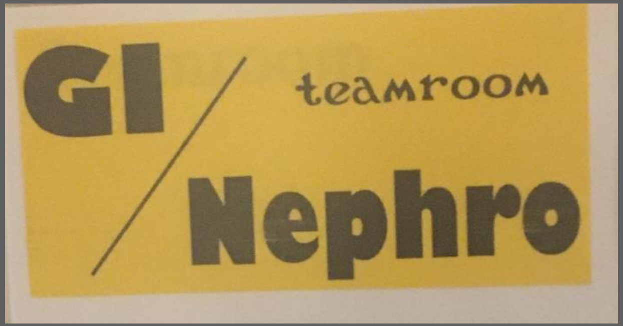
GI
teamroom
Nephro

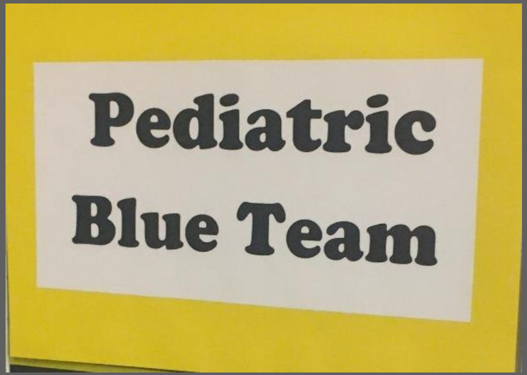
Pediatric
Blue Team

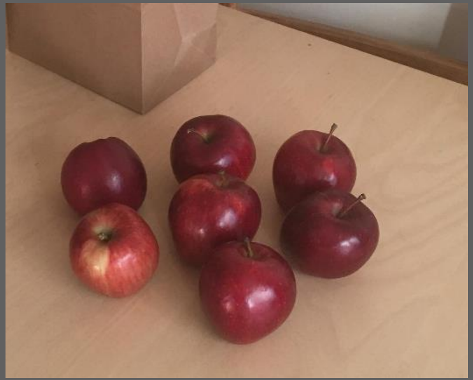

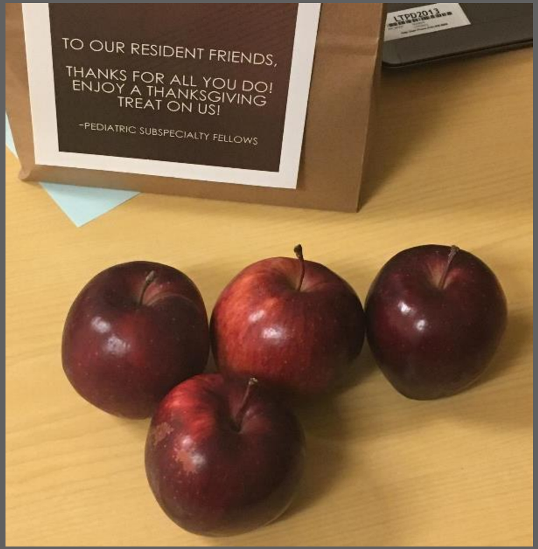
TO OUR RESIDENT FRIENDS,
THANKS FOR ALL YOU DO!
ENJOY A THANKSGIVING
TREAT ON US!
-PEDIATRIC SUBSPECIALTY FELLOWS

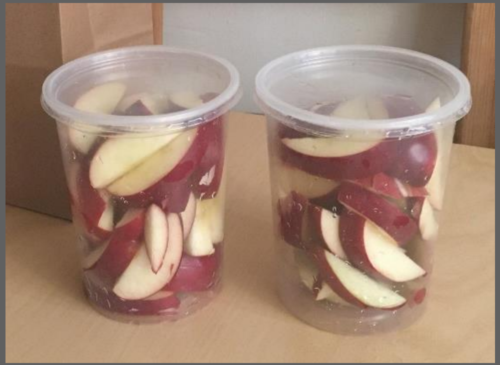

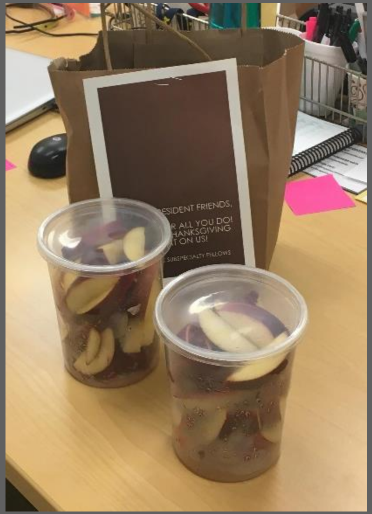

POPCORN

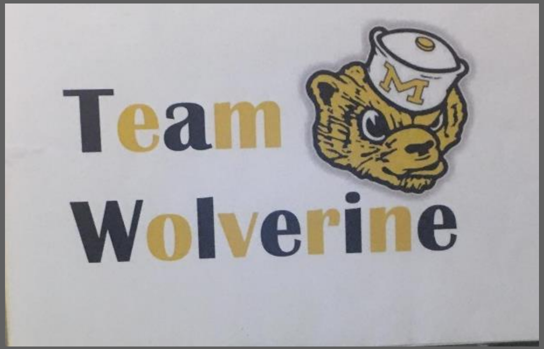
Team
Wolverine

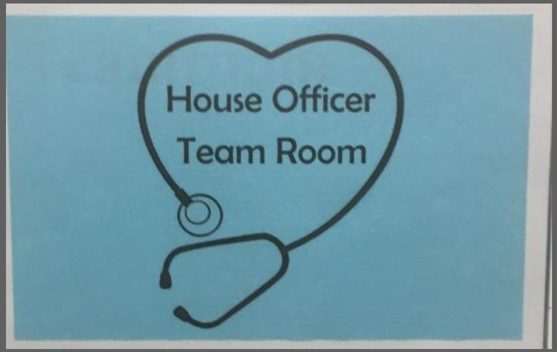
House Officer
Team Room

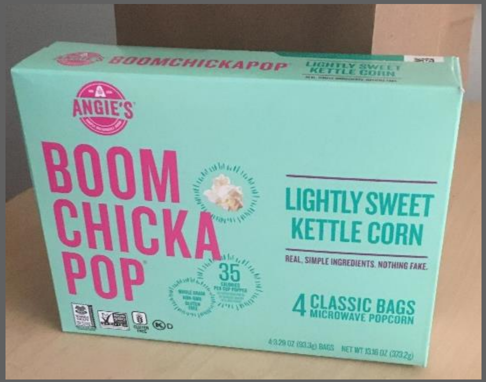
ANGIE'S
BOOM
CHICKA
POP
LIGHTLY SWEET
KETTLE CORN
REAL, SIMPLE INGREDIENTS. NOTHING FAKE.
4 CLASSIC BAGS
MICROWAVE POPCORN

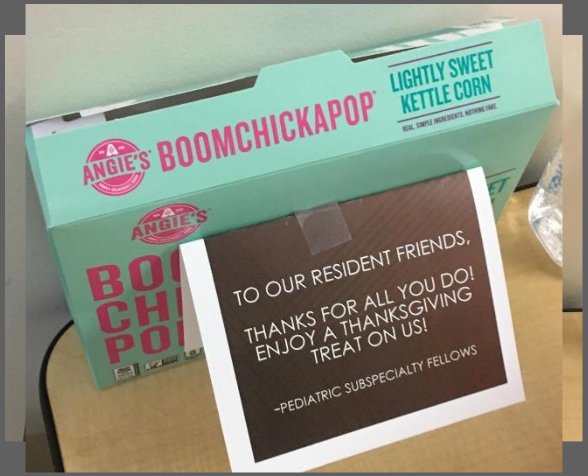
ANGIE'S BOOMCHICKAPOP
LIGHTLY SWEET
KETTLE CORN
TO OUR RESIDENT FRIENDS,
THANKS FOR ALL YOU DO!
ENJOY A THANKSGIVING
TREAT ON US!
-PEDIATRIC SUBSPECIALTY FELLOWS

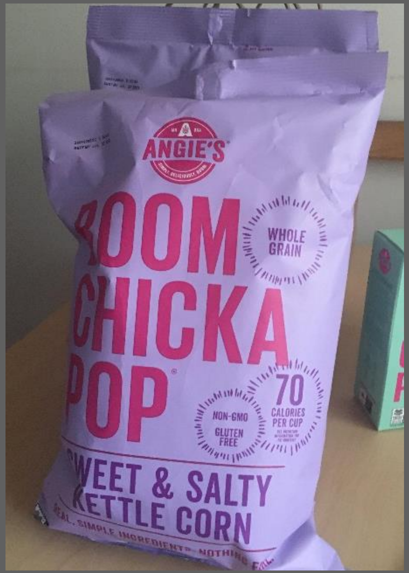
ANGIE'S
CHICKA
POP
WHOLE
GRAIN
70
CALORIES
PER CUP
NON-GMO
GLUTEN
FREE
KETTLE CORN

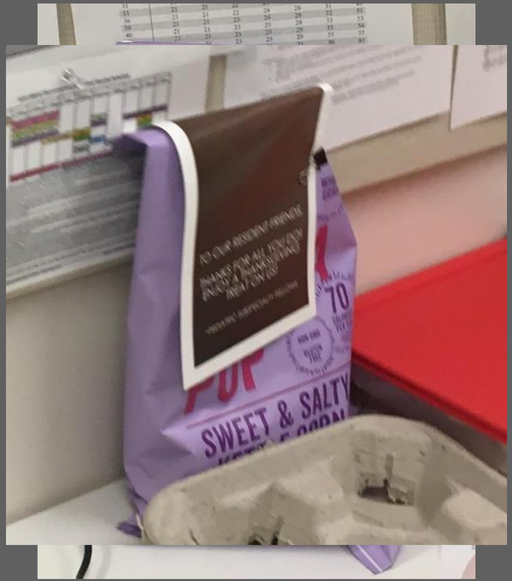
SWEET & SALTY

M&M'S

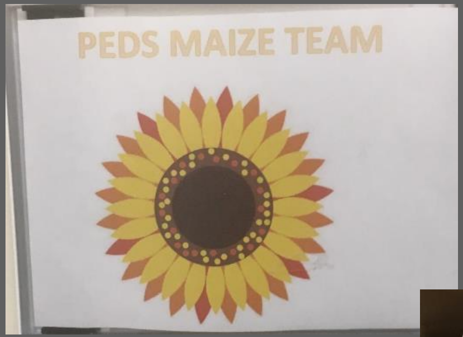
PEDS MAIZE TEAM

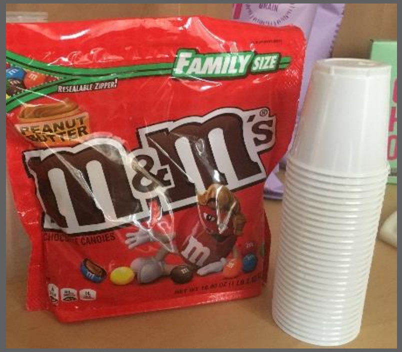
FAMILY SIZE
PEANUT BUTTER
m&m's

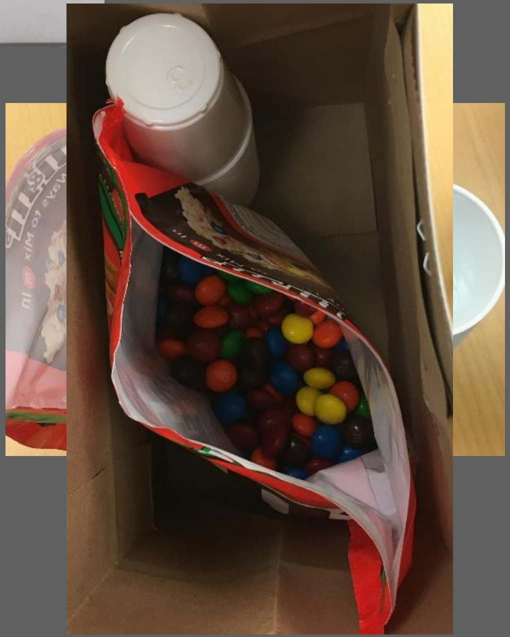

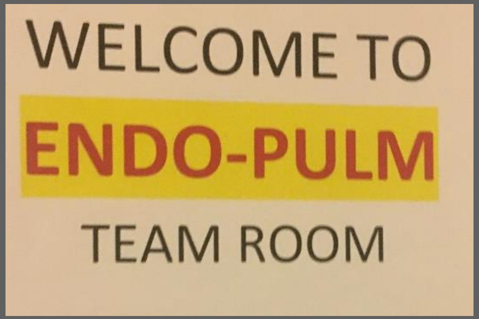
WELCOME TO
ENDO-PULM
TEAM ROOM

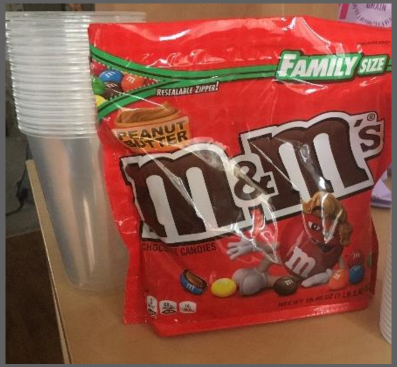
FAMILY SIZE
PEANUT BUTTER
m&m's

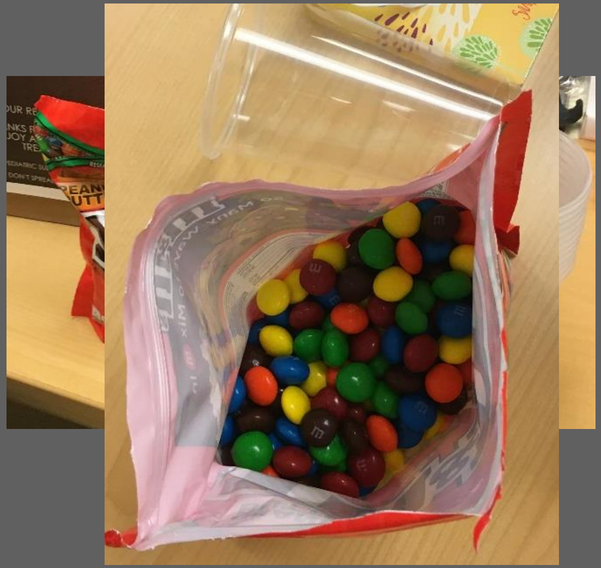

OUR ENVIRONMENT PLAYS A BIG ROLE IN HEALTHY HABITS

Take-Away Point #1

HIDDEN BRAIN-HABITS

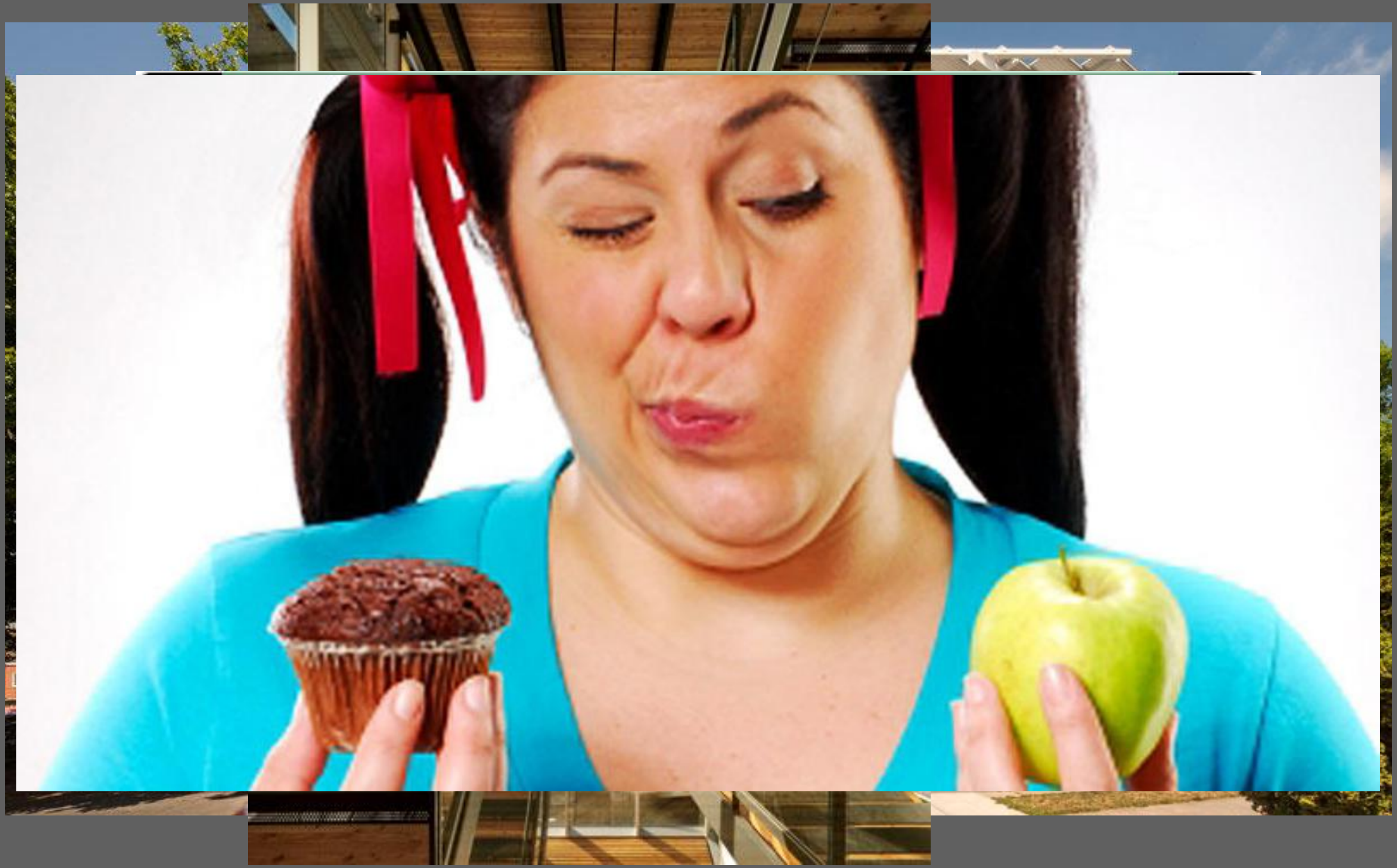

HOW CAN YOU BE IN CONTROL?

- Make ½ your plate fruits and vegetables
- Use a smaller plate
- Don’t clear your plate
- Don’t let the workers add fats and salts
- Ask a restaurant to put ½ your food in a to go container before bringing it
- Reconsider your breakfast

BE IN CONTROL BY:
RECONSIDERING YOUR BREAKFAST

- AM BS ~80
- Average American breakfast will make your blood sugar spike (~200-300)
- You then crash to 60's AROUND 10-11
- Pick a food for Bfast that will help you feel full
  - Protein or whole grain
    - Fruit, Eggs, PB, Protein bars, meat
- Don't skip breakfast

BE IN CONTROL BY:
KNOWING YOUR PORTIONS

BE IN CONTROL BY:
CHOOSING BETTER SNACKS

- Make sure you have readily available healthy options
  - Consider low fat dairy products
  - When cooking, cut up an extra apple, banana, carrot, celery, etc for your snacks
- Measure out an amount
  - Don’t take the whole bag to the couch
- Carry your own snacks instead of relying on what is laying around the office.
  - Consider what will make/keep you full

CHANGING HABITS

WITH THE HELP OF MYPLATE

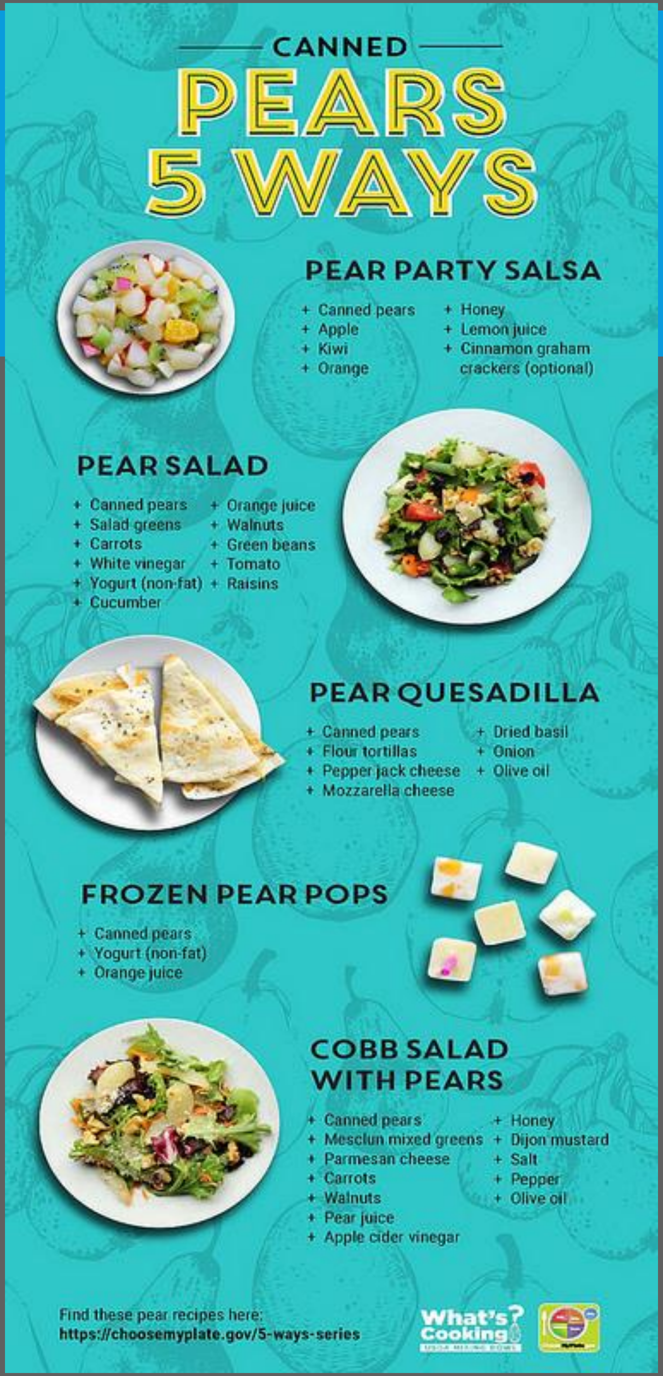

CANNED
PEARS
5 WAYS
PEAR PARTY SALSA
+ Canned pears
+ Apple
+ Kiwi
+ Orange
+ Honey
+ Lemon juice
+ Cinnamon graham crackers (optional)
PEAR SALAD
+ Canned pears
+ Salad greens
+ Carrots
+ White vinegar
+ Yogurt (non-fat)
+ Cucumber
+ Orange juice
+ Walnuts
+ Green beans
+ Tomato
+ Raisins
PEAR QUESADILLA
+ Canned pears
+ Flour tortillas
+ Pepper jack cheese
+ Mozzarella cheese
+ Dried basil
+ Onion
+ Olive oil
FROZEN PEAR POPS
+ Canned pears
+ Yogurt (non-fat)
+ Orange juice
COBB SALAD WITH PEARS
+ Canned pears
+ Mesclun mixed greens
+ Parmesan cheese
+ Carrots
+ Walnuts
+ Pear juice
+ Apple cider vinegar
+ Honey
+ Dijon mustard
+ Salt
+ Pepper
+ Olive oil
Find these pear recipes here:
https://choosemyplate.gov/5-ways-series
What's Cooking?

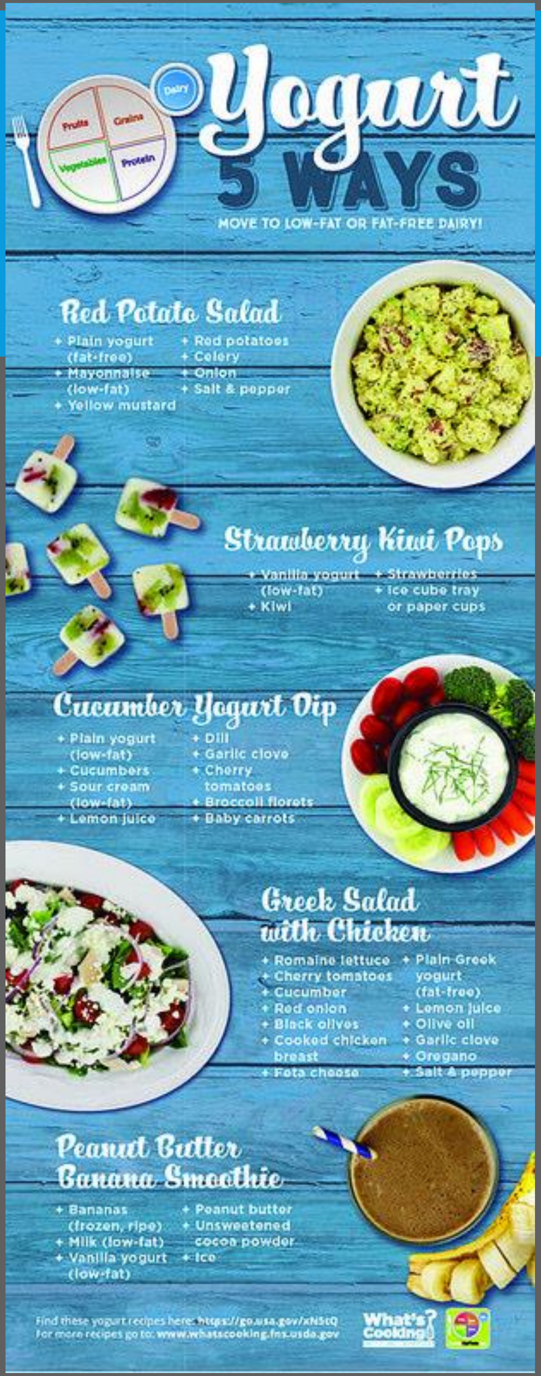

Fruits
Grains
Vegetables
Protein
Dairy
Yogurt
5 WAYS
MOVE TO LOW-FAT OR FAT-FREE DAIRY!
Red Potato Salad
+ Plain yogurt (fat-free)
+ Mayonnaise (low-fat)
+ Yellow mustard
+ Red potatoes
+ Celery
+ Onion
+ Salt & pepper
Strawberry Kiwi Pops
+ Vanilla yogurt (low-fat)
+ Kiwi
+ Strawberries
+ Ice cube tray or paper cups
Cucumber Yogurt Dip
+ Plain yogurt (low-fat)
+ Cucumbers
+ Sour cream (low-fat)
+ Lemon juice
+ Dill
+ Garlic clove
+ Cherry tomatoes
+ Broccoli florets
+ Baby carrots
Greek Salad with Chicken
+ Romaine lettuce
+ Cherry tomatoes
+ Cucumber
+ Red onion
+ Black olives
+ Cooked chicken breast
+ Feta cheese
+ Plain Greek yogurt (fat-free)
+ Lemon juice
+ Olive oil
+ Garlic clove
+ Oregano
+ Salt & pepper
Peanut Butter Banana Smoothie
+ Bananas (frozen, ripe)
+ Milk (low-fat)
+ Vanilla yogurt (low-fat)
+ Peanut butter
+ Unsweetened cocoa powder
+ Ice
Find these yogurt recipes here: https://go.usa.gov/xN5tQ
For more recipes go to: www.whatscooking.fns.usda.gov
What's Cooking?

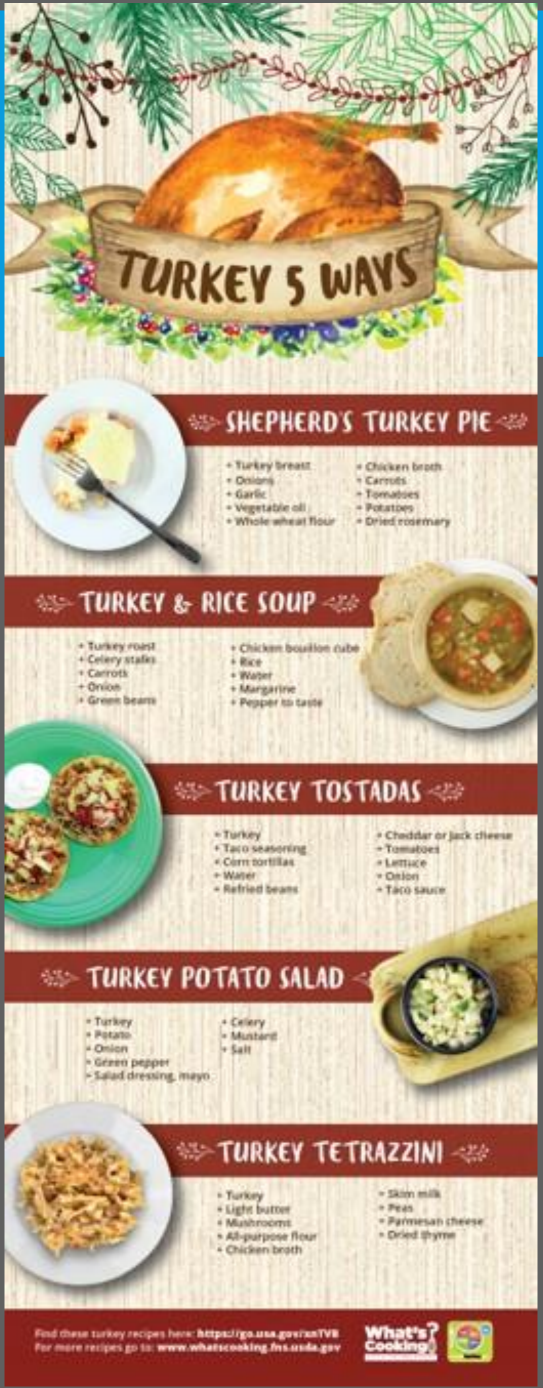

TURKEY 5 WAYS
SHEPHERD'S TURKEY PIE
+ Turkey breast
+ Onions
+ Garlic
+ Vegetable oil
+ Whole wheat flour
+ Chicken broth
+ Carrots
+ Tomatoes
+ Potatoes
+ Dried rosemary
TURKEY & RICE SOUP
+ Turkey roast
+ Celery stalks
+ Carrots
+ Onion
+ Green beans
+ Chicken bouillon cube
+ Rice
+ Water
+ Margarine
+ Pepper to taste
TURKEY TOSTADAS
+ Turkey
+ Taco seasoning
+ Corn tortillas
+ Water
+ Refried beans
+ Cheddar or jack cheese
+ Tomatoes
+ Lettuce
+ Onion
+ Taco sauce
TURKEY POTATO SALAD
+ Turkey
+ Potato
+ Onion
+ Green pepper
+ Salad dressing, mayo
+ Celery
+ Mustard
+ Salt
TURKEY TETRAZZINI
+ Turkey
+ Light butter
+ Mushrooms
+ All-purpose flour
+ Chicken broth
+ Skim milk
+ Peas
+ Parmesan cheese
+ Dried thyme
Find these turkey recipes here: https://go.usa.gov/xnTV8
For more recipes go to: www.whatscooking.fns.usda.gov
What's Cooking?

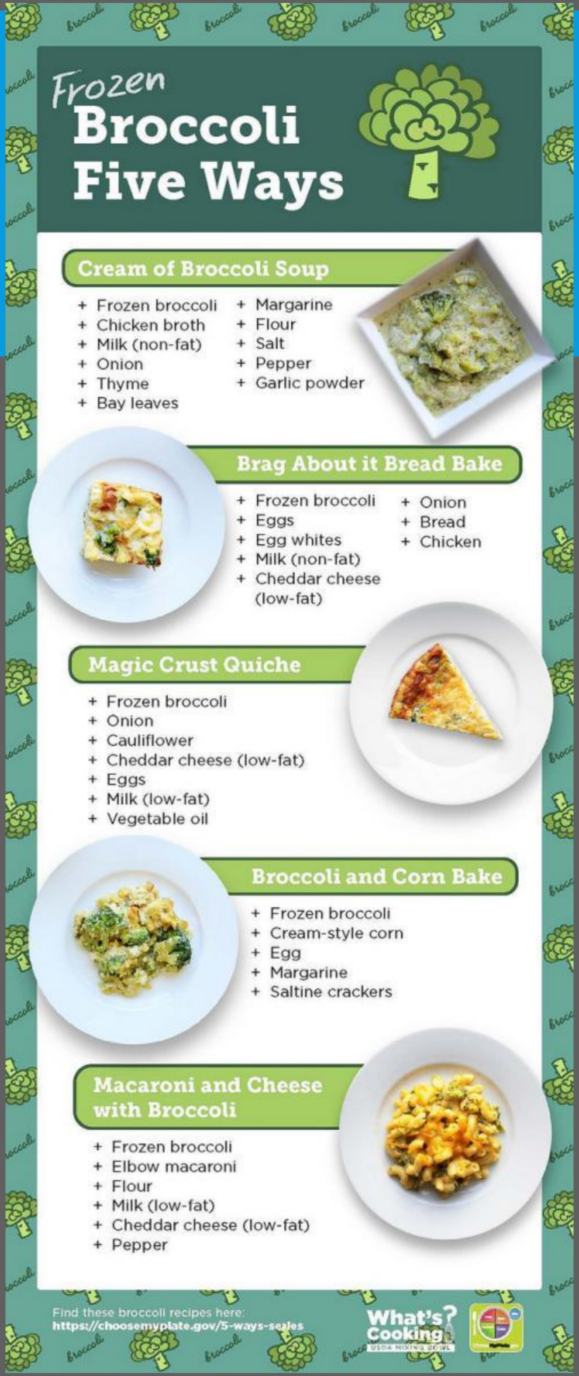

Frozen
Broccoli
Five Ways
Cream of Broccoli Soup
+ Frozen broccoli
+ Chicken broth
+ Milk (non-fat)
+ Onion
+ Thyme
+ Bay leaves
+ Margarine
+ Flour
+ Salt
+ Pepper
+ Garlic powder
Brag About it Bread Bake
+ Frozen broccoli
+ Eggs
+ Egg whites
+ Milk (non-fat)
+ Cheddar cheese (low-fat)
+ Onion
+ Bread
+ Chicken
Magic Crust Quiche
+ Frozen broccoli
+ Onion
+ Cauliflower
+ Cheddar cheese (low-fat)
+ Eggs
+ Milk (low-fat)
+ Vegetable oil
Broccoli and Corn Bake
+ Frozen broccoli
+ Cream-style corn
+ Egg
+ Margarine
+ Saltine crackers
Macaroni and Cheese with Broccoli
+ Frozen broccoli
+ Elbow macaroni
+ Flour
+ Milk (low-fat)
+ Cheddar cheese (low-fat)
+ Pepper
Find these broccoli recipes here:
https://choosemyplate.gov/5-ways-series
What's Cooking?

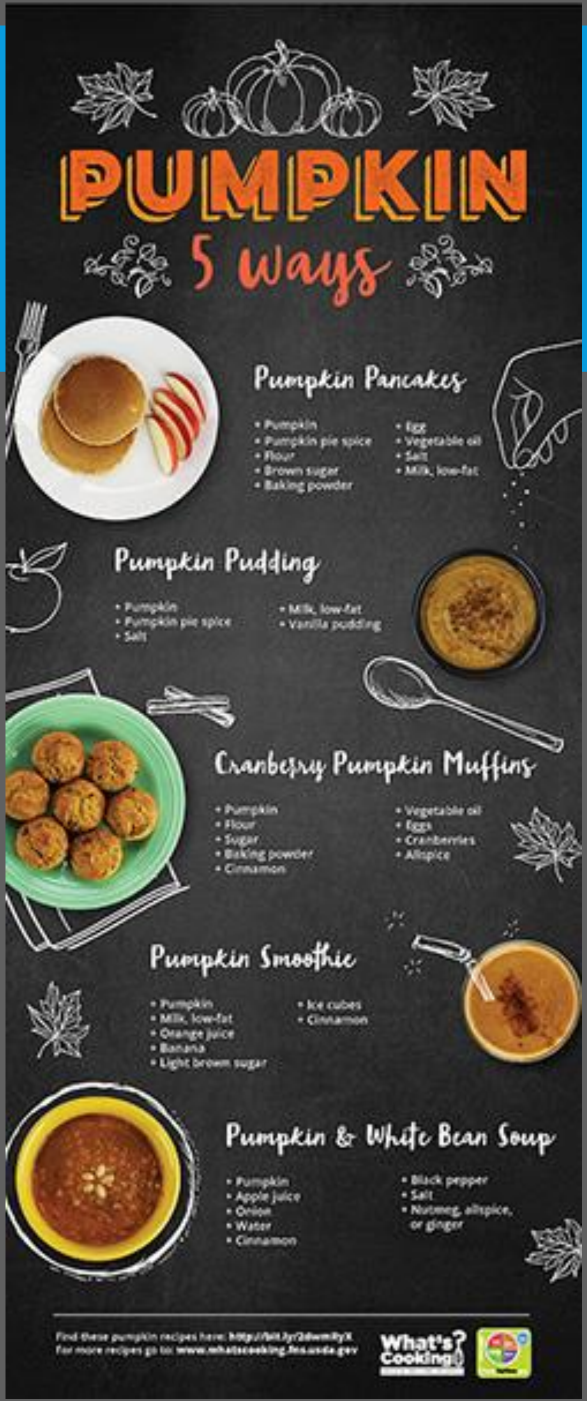
PUMPKIN
5 ways
Pumpkin Pancakes
• Pumpkin
• Pumpkin pie spice
• Flour
• Brown sugar
• Baking powder
• Egg
• Vegetable oil
• Salt
• Milk, low-fat
Pumpkin Pudding
• Pumpkin
• Pumpkin pie spice
• Salt
• Milk, low-fat
• Vanilla pudding
Cranberry Pumpkin Muffins
• Pumpkin
• Flour
• Sugar
• Baking powder
• Cinnamon
• Vegetable oil
• Eggs
• Cranberries
• Allspice
Pumpkin Smoothie
• Pumpkin
• Milk, low-fat
• Orange juice
• Banana
• Light brown sugar
• Ice cubes
• Cinnamon
Pumpkin & White Bean Soup
• Pumpkin
• Apple juice
• Onion
• Water
• Cinnamon
• Black pepper
• Salt
• Nutmeg, allspice, or ginger
For more recipes go to: www.whatscooking.fns.usda.gov
What's Cooking

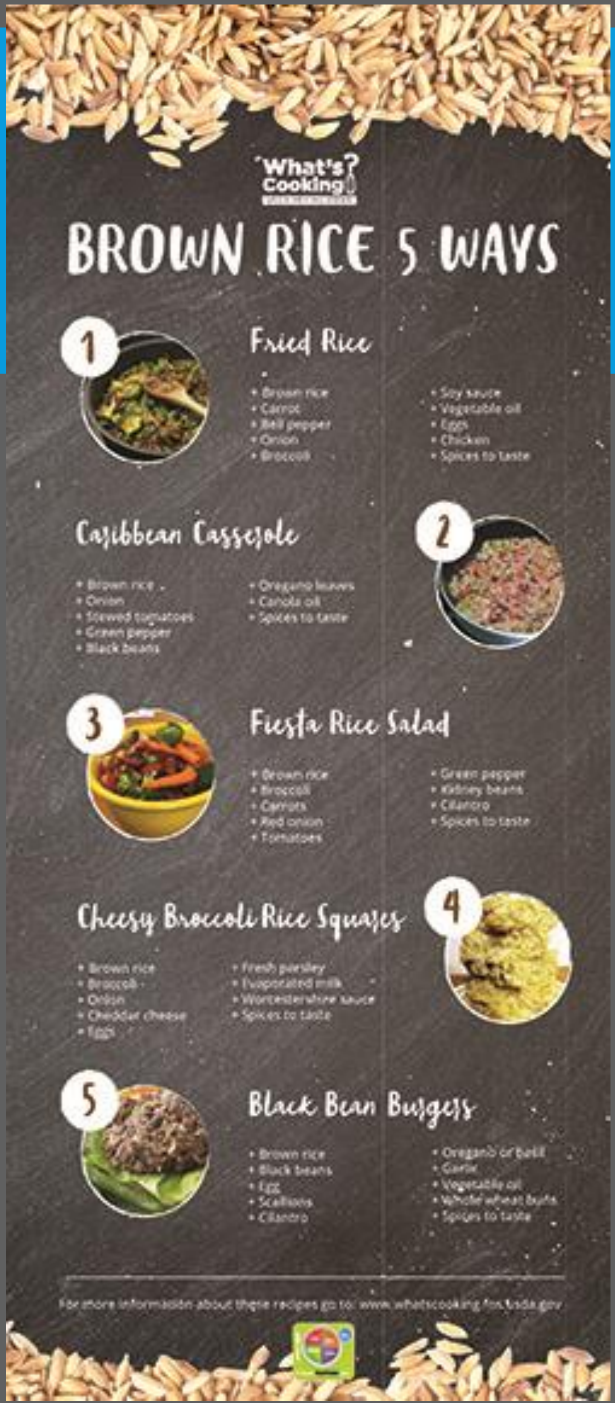
What's Cooking
BROWN RICE 5 WAYS
1
Fried Rice
• Brown rice
• Carrot
• Bell pepper
• Onion
• Broccoli
• Soy sauce
• Vegetable oil
• Eggs
• Chicken
• Spices to taste
Caribbean Casserole
2
• Brown rice
• Onion
• Stewed tomatoes
• Green pepper
• Black beans
• Oregano leaves
• Canola oil
• Spices to taste
3
Fiesta Rice Salad
• Brown rice
• Broccoli
• Carrots
• Red onion
• Tomatoes
• Green pepper
• Kidney beans
• Cilantro
• Spices to taste
Cheesy Broccoli Rice Squares
4
• Brown rice
• Broccoli
• Onion
• Cheddar cheese
• Eggs
• Fresh parsley
• Evaporated milk
• Worcestershire sauce
• Spices to taste
5
Black Bean Burgers
• Brown rice
• Black beans
• Egg
• Scallions
• Cilantro
• Oregano or basil
• Garlic
• Vegetable oil
• Whole wheat buns
• Spices to taste
For more information about these recipes go to: www.whatscooking.fns.usda.gov

MyPlate MyWins

Hacking your snacks

Planning for healthy snacks can help satisfy hunger in between meals and keep you moving towards your food group goals.

Build your own

Make your own trail mix with unsalted nuts and add-ins such as seeds, dried fruit, popcorn, or a sprinkle of chocolate chips.

Prep ahead

Portion snack foods into baggies or containers when you get home from the store so they're ready to grab-n-go when you need them.

Make it a combo

Combine food groups for a satisfying snack—yogurt and berries, apple with peanut butter, whole-grain crackers with turkey and avocado.

Eat vibrant veggies

Spice up raw vegetables with dips. Try dipping bell peppers, carrots, or cucumbers in hummus, tzatziki, guacamole, or baba ganoush.

Snack on the go

Bring ready-to-eat snacks when you're out. A banana, yogurt (in a cooler), or baby carrots are easy to bring along and healthy options.

List more tips

MyPlate MyWins

Enjoy Italian cuisine

Savor your favorite Italian meals in a healthier way with these small changes.

Balance your options

You don't necessarily have to skip the bread, wine, and dessert. Consider choosing just one of these options and pass on refills.

Start with salad

Choose a mixed green salad with vinaigrette instead of fried appetizers, which are higher in sodium and saturated fat.

Go for whole grains

Ask for whole-wheat pasta or pizza crust. Whole grains have more fiber and nutrients than refined grains.

Lighten up on cheese

Ask for a lighter sprinkling of cheese on pizza or pasta to cut back on the saturated fat and sodium.

Choose red sauces

Pick sauces made from vegetables, like marinara sauce, rather than heavy cream or butter sauce.

List more tips

MyPlate MyWins

Reach your nutrition goals

To help you achieve your nutrition goals, try using the tips below.

Start with small changes

Instead of a diet overhaul, make small changes to what you eat and drink that will work for you now and in the future.

Take one day at a time

Sometimes things don't go as planned, even with the best of intentions. If you miss one day or one milestone for your goal, don't give up!

Be active your way

Pick activities you enjoy! If you focus on having fun or learning a new skill that interests you, you will be more likely to stick with it.

Team up

Find a friend with similar goals—swap healthy recipes and be active together. Staying on track is easier with support and a cheerleader.

Celebrate successes

Think of each change as a "win" as you build positive habits and find ways to reach your goals. Reward yourself—you've earned it!

List more tips

MYPLATE IDEAS

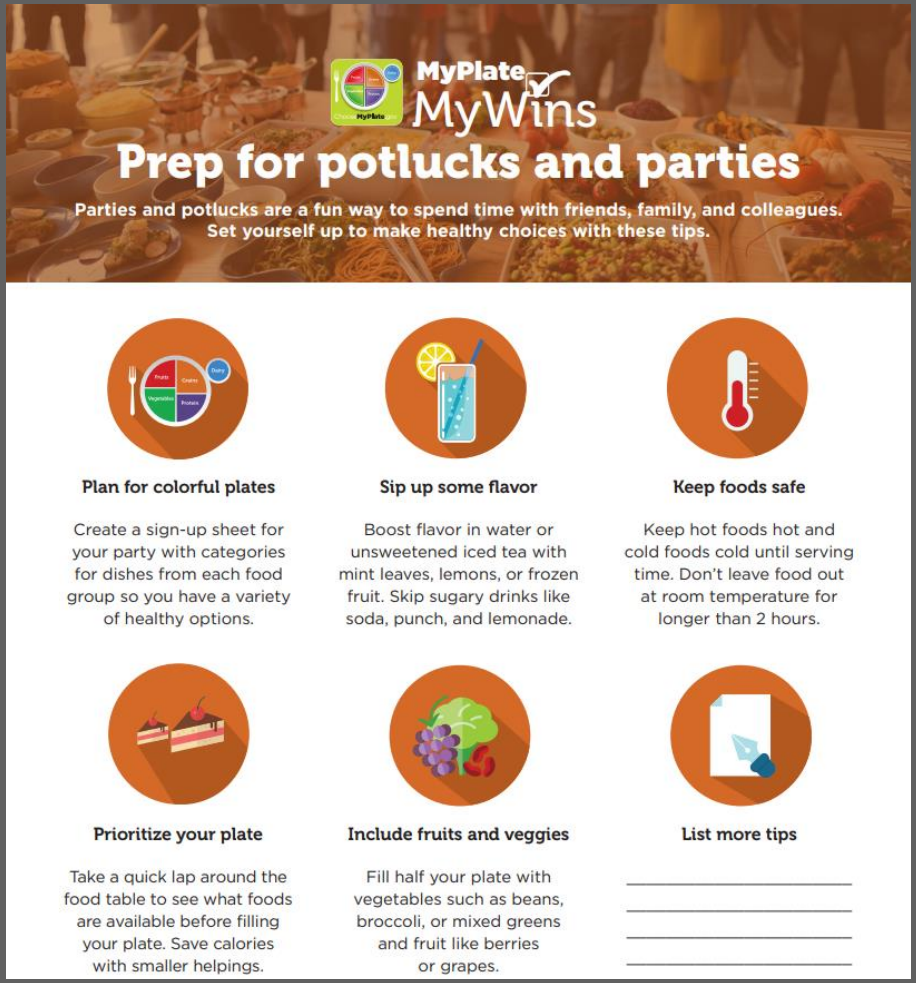

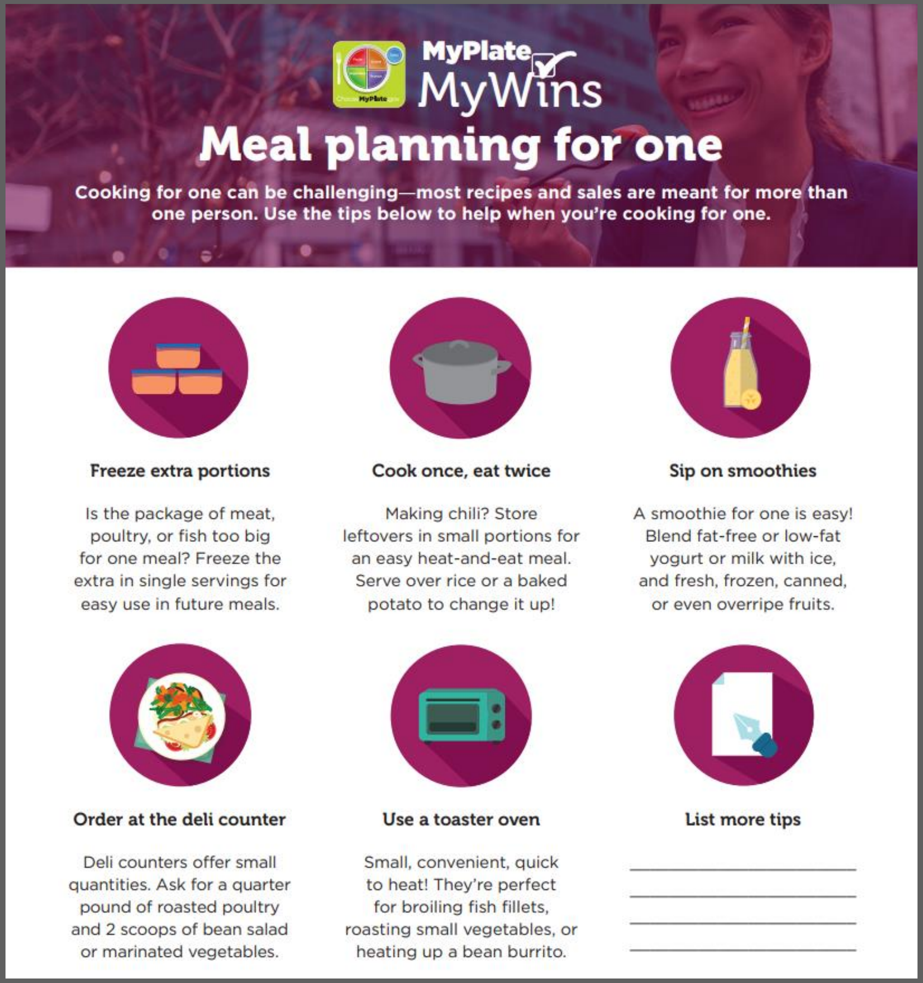

10 tips Nutrition Education Series

MyPlate MyWins

Based on the Dietary Guidelines for Americans

Build a healthy meal

Each meal is a building block in your healthy eating style. Make sure to include all the food groups throughout the day. Make fruits, vegetables, grains, dairy, and protein foods part of your daily meals and snacks. Also, limit added sugars, saturated fat, and sodium. Use the MyPlate Daily Checklist and the tips below to meet your needs throughout the day.

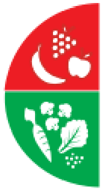

1 Make half your plate veggies and fruits
Vegetables and fruits are full of nutrients that support good health. Choose fruits and red, orange, and dark-green vegetables such as tomatoes, sweet potatoes, and broccoli.

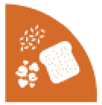

2 Include whole grains
Aim to make at least half your grains whole grains. Look for the words “100% whole grain” or “100% whole wheat” on the food label. Whole grains provide more nutrients, like fiber, than refined grains.

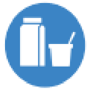

3 Don’t forget the dairy
Complete your meal with a cup of fat-free or low-fat milk. You will get the same amount of calcium and other essential nutrients as whole milk but fewer calories. Don’t drink milk? Try a soy beverage (soymilk) as your drink or include low-fat yogurt in your meal or snack.

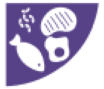

4 Add lean protein
Choose protein foods such as lean beef, pork, chicken, or turkey, and eggs, nuts, beans, or tofu. Twice a week, make seafood the protein on your plate.

5 Avoid extra fat
Using heavy gravies or sauces will add fat and calories to otherwise healthy choices. Try steamed broccoli with a sprinkling of low-fat parmesan cheese or a squeeze of lemon.

6 Get creative in the kitchen
Whether you are making a sandwich, a stir-fry, or a casserole, find ways to make them healthier. Try using less meat and cheese, which can be higher in saturated fat and sodium, and adding in more veggies that add new flavors and textures to your meals.

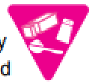

7 Take control of your food
Eat at home more often so you know exactly what you are eating. If you eat out, check and compare the nutrition information. Choose options that are lower in calories, saturated fat, and sodium.

8 Try new foods
Keep it interesting by picking out new foods you’ve never tried before, like mango, lentils, quinoa, kale, or sardines. You may find a new favorite! Trade fun and tasty recipes with friends or find them online.

9 Satisfy your sweet tooth in a healthy way
Indulge in a naturally sweet dessert dish—fruit! Serve a fresh fruit salad or a fruit parfait made with yogurt. For a hot dessert, bake apples and top with cinnamon.

10 Everything you eat and drink matters
The right mix of foods in your meals and snacks can help you be healthier now and into the future. Turn small changes in how you eat into your MyPlate, MyWins.

10 tips Nutrition Education Series

MyPlate MyWins

Based on the Dietary Guidelines for Americans

Add more vegetables to your day

It’s easy to eat more vegetables! Eating vegetables is important because they provide vitamins and minerals and most are low in calories. To fit more vegetables in your day, try them as snacks and add them to your meals.

1 Discover fast ways to cook
Cook fresh or frozen vegetables in the microwave for a quick-and-easy dish to add to any meal. Steam green beans, carrots, or bok choy in a bowl with a small amount of water in the microwave for a quick side dish.

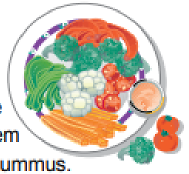

2 Be ahead of the game
Cut up a batch of bell peppers, cauliflower, or broccoli. Pre-package them to use when time is limited. Enjoy them in a casserole, stir-fry, or as a snack with hummus.

3 Choose vegetables rich in color
Brighten your plate with vegetables that are red, orange, or dark green. They are full of vitamins and minerals. Try acorn squash, cherry tomatoes, sweet potatoes, or collard greens. They not only taste great but are good for you, too.

4 Check the freezer aisle
Frozen vegetables are quick and easy to use and are just as nutritious as fresh veggies. Try adding frozen vegetables, such as corn, peas, edamame, or spinach, to your favorite dish. Look for frozen vegetables without added sauces, gravies, butter, or cream.

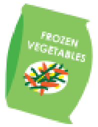

5 Stock up on veggies
Canned vegetables are a great addition to any meal, so keep on hand canned tomatoes, kidney beans, garbanzo beans, mushrooms, and beets. Select those labeled as “reduced sodium,” “low sodium,” or “no salt added.”

6 Make your garden salad glow with color
Brighten your salad by using colorful vegetables such as black beans or avocados, sliced red bell peppers or onions, shredded radishes or carrots, and chopped red cabbage or watercress. Your salad will not only look good but taste good, too.

7 Sip on some vegetable soup
Heat it and eat it. Try tomato, butternut squash, or garden vegetable soup. Look for reduced- or low-sodium soups. Make your own soups with a low-sodium broth and your favorite vegetables.

8 While you’re out
If dinner is away from home, no need to worry. When ordering, ask for an extra side of vegetables or a side salad instead of the typical fried side dish. Ask for toppings and dressings on the side.

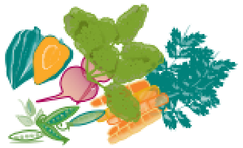

9 Savor the flavor of seasonal vegetables
Buy vegetables that are in season for maximum flavor at a lower cost. Check your local supermarket specials for the best in-season buys. Or visit your local farmers market.

10 Vary your veggies
Choose a new vegetable that you’ve never tried before. Find recipes online at WhatsCooking.fns.usda.gov.

"UP" YOUR ACTIVITY

IT ALL *ADDS UP*

- Pay attention to your pedometer
  - Just recording steps/kCal leads to healthier choices
    - Do a weekly check-in
    - Weight, steps, kCals, etc
- Use a printer farther away
- Walk 2 flights of stairs
- Stand for every phone call
- 7 min workouts
- Consider home gym equipment

TEAM UP FOR SUCCESS

- Healthy outcomes are improved when you work with others
  - Get a resident buddy to do things with
  - Don't judge each other
- Set team goals. Ideas may include:
  - Healthy snack of the day/week
  - Team stairs
  - Water goals
  - Pedometer competition

ONE UP YOURSELF

Add the score from page 1 __________ to the score on this page __________ = __________

IF YOUR SCORE IS:

19-29	There are MANY ways you can make your eating pattern more healthy.
29-43	There are SOME ways you can make your eating pattern more healthy.
43-57	You are making MANY healthy choices.

Eastern Carolina Family Practice Center 12/98

ONE UP YOURSELF

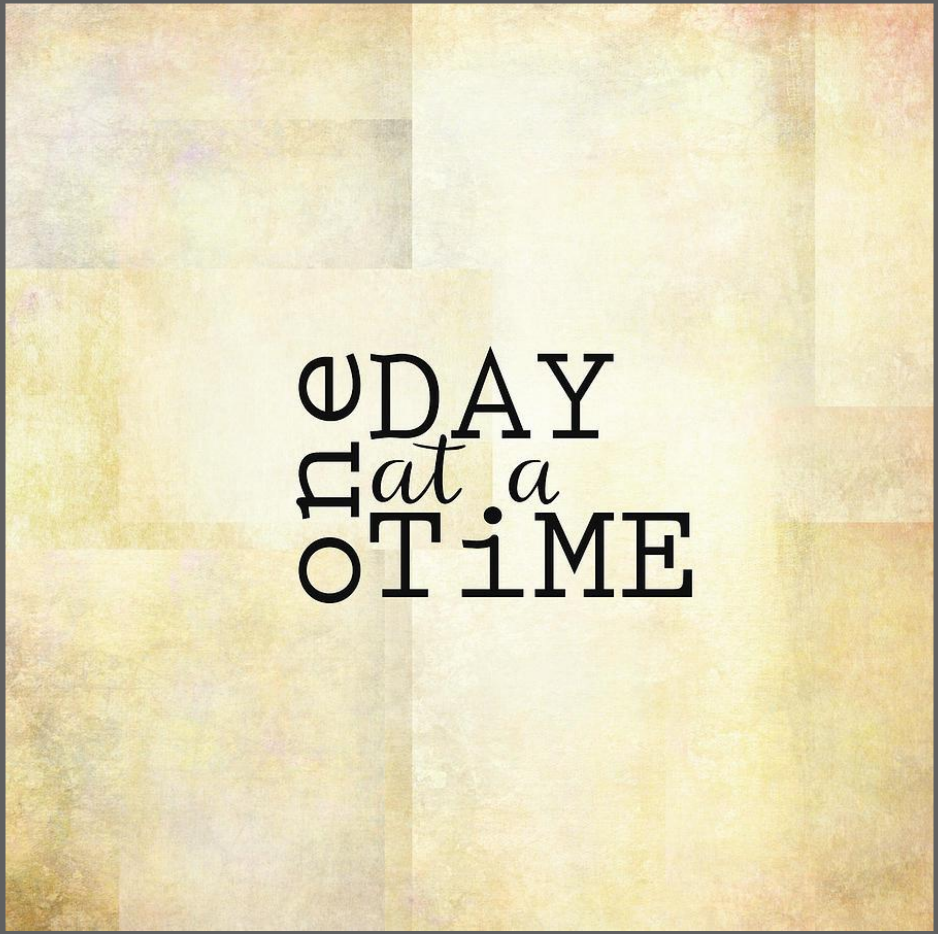

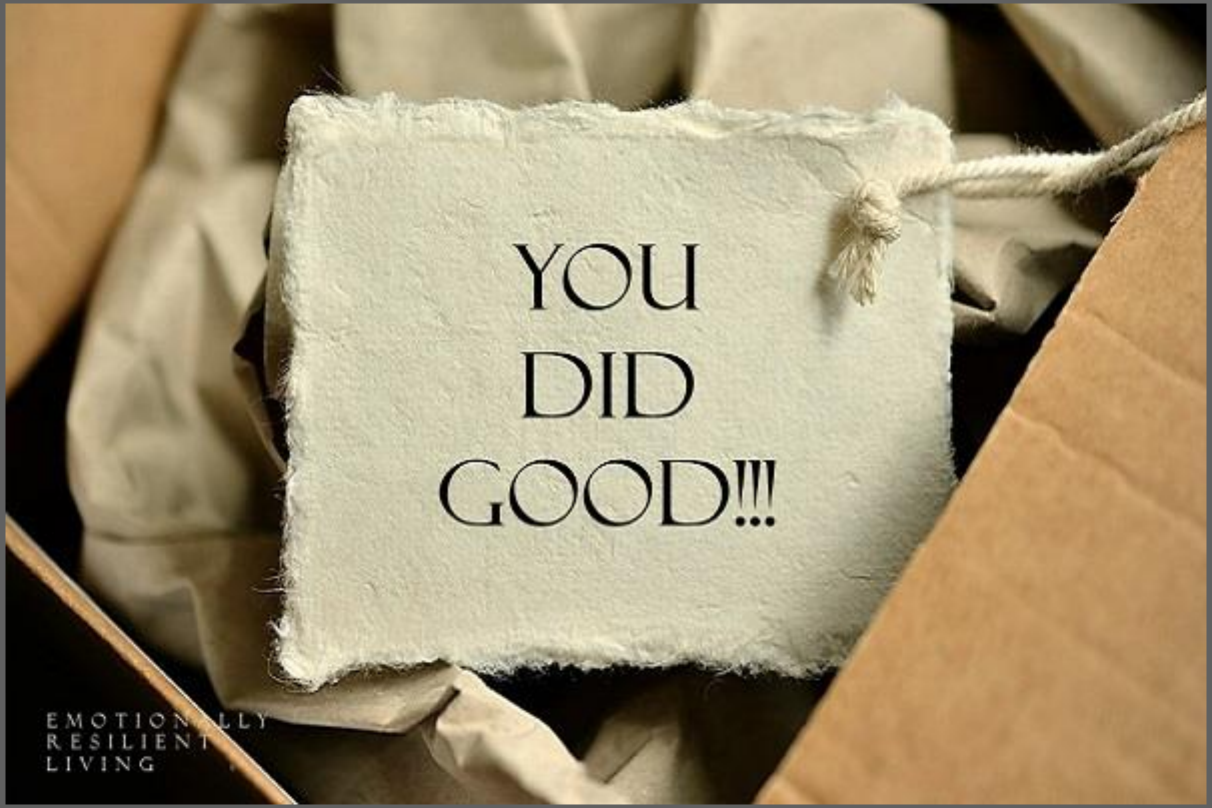

QUESTIONS?
