# Supplementary material for: Application of an Interactive, Hands-On Nutritional Curriculum for Pediatric Residents
Source: JPGN Rep. 2023 Nov 13;4(4):e384. doi: 10.1097/PG9.0000000000000384 (PMC10684231; doi:10.1097/PG9.0000000000000384)
Supplement: Supplementary file 9 [file pg9-4-e384-s009.pdf]

Please rate your comfort level performing each of the following in the outpatient setting:

	Very Uncomfortable	Somewhat uncomfortable	Neither comfortable nor uncomfortable	Somewhat comfortable	Very comfortable
Finding clinical resources and references on the USDA MyPlate website	○	○	○	○	○
Referring my patients to high quality digital content to meet their weight loss and fitness goals	○	○	○	○	○
Evaluating the safety and efficacy of weight loss programs, dietary supplements, and meal replacement products using evidence-based principles and resources	○	○	○	○	○
Providing nutritional strategies to patients trying to lose weight	○	○	○	○	○

Page Break

Please select the risk factors for obesity. Choose all correct answers.

- ☐ Parental BMI
- ☐ Infant Birth Weight
- ☐ >2 hrs per day of screen time
- ☐ <8 hours of sleep
- ☐ Two parent household
- ☐ Participation in WIC
- ☐ Intake of sugar sweetened beverages
- ☐ Single parent household
- ☐ Fat content of milk
- ☐ Parental knowledge of the nutrition facts label

In which settings is BMI appropriate to use. Please choose all that apply.

- ☐ In children <2 yo
- ☐ In children 2-5 yo
- ☐ In children >5 yo
- ☐ In weightlifters
- ☐ As a measure of total body fat
- ☐ To predict cardiovascular disease in a population
- ☐ To predict cardiovascular disease in an individual

I regularly counsel about the nutritional density of given foods using the following. Please choose all that apply.

- ☐ Caloric Content
- ☐ Sodium Content
- ☐ Added Sugar Content
- ☐ Fat Content
- ☐ Ingredients List
- ☐ Fiber Content

Page Break

How often do you do the following during well child exams?

	Always	Most of the time	About half the time	Rarely	Never
Evaluate BMI	○	○	○	○	○
Evaluate diet	○	○	○	○	○
Evaluate level of physical activity	○	○	○	○	○
Assess readiness for improvement in diet or physical activity	○	○	○	○	○
Make recommendations for dietary improvement	○	○	○	○	○
Make recommendations for physical activity improvement	○	○	○	○	○

True/False: I consider an improvement in my patient's ________ a requirement to successful counseling on diet and physical activity.

	True	False
BMI	○	○
A1C	○	○
Blood Pressure	○	○

I consider my dietary and/or physical activity counseling effective if...(Please select all that apply)

	Much better	Moderately better	Slightly better	About the same	Worse
My patient and/or family attitude toward healthy eating are..	☐	☐	☐	☐	☐
My patient and/or family attitude toward physical activity are...	☐	☐	☐	☐	☐
My patient and/or family understanding of healthy diet/activity are...	☐	☐	☐	☐	☐
Dietary habits are..	☐	☐	☐	☐	☐
Physical activity habits are...	☐	☐	☐	☐	☐
